# α-Amino Acids as Reducing and Capping Agents in Gold Nanoparticles Synthesis Using the Turkevich Method

**DOI:** 10.1021/acs.langmuir.3c00507

**Published:** 2023-06-14

**Authors:** Aleksandra M. Figat, Bartosz Bartosewicz, Malwina Liszewska, Bogusław Budner, Małgorzata Norek, Bartłomiej J. Jankiewicz

**Affiliations:** † Institute of Optoelectronics, Military University of Technology, gen. Sylwestra Kaliskiego 2, 00-908 Warsaw, Poland; ‡ Faculty of Advanced Technologies and Chemistry, Military University of Technology, gen. Sylwestra Kaliskiego 2, 00-908 Warsaw, Poland

## Abstract

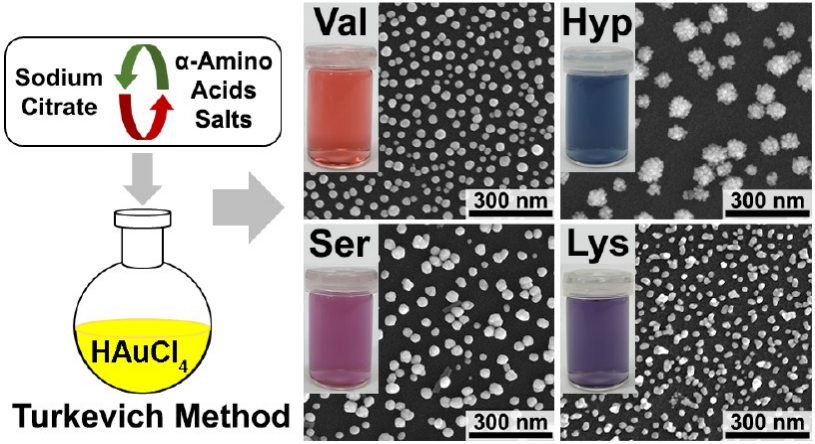

Amino acid-capped gold nanoparticles (AuNPs) are a promising tool for various applications, including therapeutics and diagnostics. Most often, amino acids are used to cap AuNPs synthesized with other reducing agents. However, only a few studies have been dedicated to using α-amino acids as reducing and capping agents in AuNPs synthesis. Hence, there are still several gaps in understanding their role in reducing gold salts. Here, we used 20 proteinogenic α-amino acids and one non-proteinogenic α-amino acid in analogy to sodium citrate as reducing and capping agents in synthesizing AuNPs using the Turkevich method. Only four of the twenty-one investigated amino acids have not yielded gold nanoparticles. The shape, size distribution, stability, and optical properties of synthesized nanoparticles were characterized by scanning electron microscopy, differential centrifugal sedimentation, the phase analysis light scattering technique, and UV–vis spectroscopy. The physicochemical characteristics of synthesized AuNPs varied with the amino acid used for the reduction. We proposed that in the initial stage of gold salts reduction most of the used α-amino acids behave similarly to citrate in the Turkevich method. However, their different physicochemical properties resulting from differences in their chemical structures significantly influence the outcomes of reactions.

## Introduction

The unique chemical and physical properties of gold nanoparticles (AuNPs) make them suitable for a broad range of applications,^1^ including biomedicine,^2^ chemical, and biological sensing,^3^ surface-enhanced Raman spectroscopy (SERS),^4^ catalysis,^5^ organic solar cells,^6^ or enhancement of photonic crystal fibers’ thermal and electro-optical properties.^7−9^ In biomedical sciences, AuNPs find applications in controlled drug delivery systems, precision therapeutics, diagnostics, and theranostics.^10−13^ AuNPs are used alone or as composites with other materials in all mentioned applications. The functional properties of AuNPs can be tailored toward specific applications by changing their size, shape, surface chemistry, or aggregation state.

The AuNPs of different sizes, shapes, and surface chemistry can be synthesized using different reagents and methodologies.^14−16^ Among the most popular protocols for synthesizing monodisperse quasi-spherical AuNPs is the reduction of chloroauric acid with citrate,^17^ most often termed the Turkevich method.^18,19^ We showed that other α-hydroxycarboxylates could also substitute citrate in this method.^20^ In recent years, growing interest has been observed in the synthesis of metal nanoparticles (NPs) using green chemistry, in which naturally occurring organisms such as bacteria, plants, algae, fungi, and yeast, as well as biomolecules produced by them, are used as both reducing and capping agents.^21−26^ The advantages of using biomolecules in synthesizing NPs include simplicity, environment-friendly nature, low cost, improved biological properties, and reduced toxicity.^26,27^ However, using green reagents, such as microbes, has some drawbacks, including difficulty in identifying the exact molecule and mechanism responsible for NPs formation and the tedious purification processes required. A similar issue is related to plant extracts, consisting of many biomolecules, although synthesis of AuNPs with plant extract is much easier than with microbes. Green synthesis of NPs has great potential, but many challenges and difficulties must be overcome to produce and apply green synthesized nanomaterials.^27,28^ Among the issues that need to be solved are low yield, nonuniform particle sizes, complex extraction procedures, and seasonal and regional availability of raw materials.^28^

The problem with control of morphology and size of NPs, and reduced stability, in green chemistry methods could be solved by better understanding the processes occurring during the reduction of metal ions and stabilization of the resulting metal NPs. A possible solution is using simple green reagents as reducing and capping agents, such as α-amino acids (α-AA).^29−57^ The advantage of using α-AA as capping agents is that a single molecule offers amino and carboxy groups for conjugating essential biomolecules. They also increase the biocompatibility of nanoparticles they cap and reduce issues associated with using them in biomedical applications, such as size, biodistribution, interaction with immune cells, and induction of inflammation.^29,30^ The α-AA have also been shown in several studies as efficient reducing agents capable of reducing gold salts in a controlled manner to obtain uniform quasi-spherical gold nanoparticles.^31−57^ The advantages of using α-AA to reduce gold salts compared to other often used reducing agents, in addition to their advantages mentioned above as capping agents, include their nontoxicity, low price, and availability. Therefore, it is not surprising that many studies have reported using one,^31−48^ a few,^49−54^ or all 20 proteinogenic α-AA for synthesizing AuNPs.^55−57^ In most of these studies, different reaction conditions have been used for different amino acids (Supporting Information, Table S1). Only in three articles were all 20 proteinogenic α-AA used for AuNPs synthesis using similar or the same reaction conditions for comparison.^55−57^ However, these studies differed in reaction conditions (concentrations of reagents, reaction temperature, and pH). In addition, the authors in none of these articles provided characterization results for all synthesized or stable AuNPs. Interestingly, in two articles, it was observed that similar to the Turkevich method, α-AA were able to reduce the gold salts in a concentration-dependent manner, with higher concentrations leading to smaller particles, and vice versa.^56,57^ In only a few articles, the authors proposed a reaction mechanism for reducing gold salts with amino acids.^31,34,38,43^ Nevertheless, the authors in none of the mentioned studies noticed the similarity in reactivity of α-amino acids toward gold salts to one of the most common reducing and capping agents, sodium citrate.

**Chart 1:**
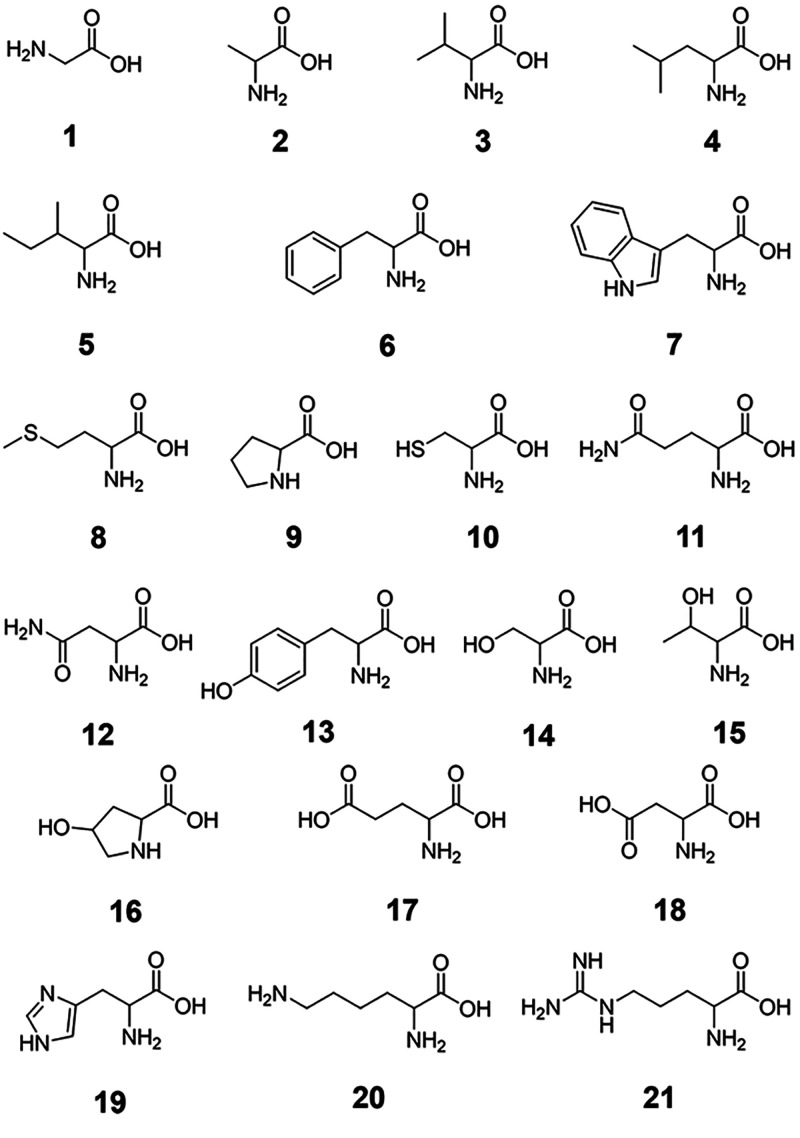
Structures of α-Amino Acids Used in Our Studies as Reducing and Capping Agents

In this article, we report the results of systematic studies on the synthesis of gold nanoparticles via the modified Turkevich method using salts of 20 proteinogenic α-amino acids, **1**–**15** and **17**–**21**, and one non-proteinogenic α-amino acid, **16** (Chart 1). The influence of α-amino acids molecular structure on the morphology, size distribution, stability, and optical properties of successfully synthesized AuNPs was investigated by using scanning electron microscopy (SEM), differential centrifugal sedimentation (DCS), phase analysis light scattering (PALS), and UV–vis spectroscopy. The results were compared to previously reported studies on synthesizing AuNPs using α-amino acids.^31−57^ Our studies also provide new insights into the mechanism of gold salt reduction by α-amino acids, and we show similarities and differences in their reactivities compared to citrate.

## Results and Discussion

### AuNPs Synthesis Using Salts of α-Amino Acids

In our studies, we used for the synthesis of AuNPs salts of twenty-one α-amino acids (Chart 1). Among the used reagents were l-glycine (Gly) (**1**), l-alanine (Ala) (**2**), l-valine (Val) (**3**), l-leucine (Leu) (**4**), l-isoleucine (Ile) (**5**), dl-phenylalanine (Phe) (**6**), l-tryptophan (Trp) (**7**), l-methionine (Met) (**8**), l-proline (Pro) (**9**), l-cysteine (Cys) (**10**), l-glutamine (Gln) (**11**), l-asparagine (Asn) (**12**), l-tyrosine (Tyr) (**13**), l-serine (Ser) (**14**), dl-threonine (Thr) (**15**), l-(4)-hydroxyproline (Hyp) (**16**), l-glutamic acid (Glu) (**17**), l-aspartic acid (Asp) (**18**), l-histidine (His) (**19**), l-lysine (Lys) (**20**), and l-arginine (Arg) (**21**). All investigated α-amino acids consist of a carboxyl group and an amine group in the α-position to it but differ in the side chain structure, giving each amino acid unique physicochemical properties (Table S2). The physicochemical properties of α-amino acids, such as solubility, stability (especially in higher temperatures), p*K*, and pI, were expected to influence the outcome of the reactions. The p*K* is particularly important because it is known that the form of gold salts and reducing agents affect the reactions’ results.^58−61^ To investigate the influence of amino acids structure on the reaction outcome, in analogy to the Turkevich method, we used salts of α-amino acids and the same reaction conditions (reaction temperature, reagents concentrations, and reaction mixture volume) in our syntheses.

Similarly to AuNPs synthesis with citrate, forming AuNPs using salts of α-amino acids requires two basic functionalities: reduction capability for metal ions and capping capability for the nanoparticles formed.^55^ It has been shown in many studies that α-amino acids can cap metal nanoparticles for their stabilization and functionalization for various applications.^29,30,62^ In most studies, metal nanoparticles were first synthesized using different reagents, and then α-AA were added to stabilize them. Stabilizing interactions of amino acids with gold nanoparticles were investigated using molecular dynamics simulations by Hoefling et al.^63^ and Ramezani et al.^64^ In the case of the latter studies, the results were compared with the experimental results and were found to be in good agreement. Based on the studies of Ramazani,^64^ Gly (**1**) is adsorbed on the AuNPs’ surface through COOH. The aliphatic amino acids having linear hydrophobic side groups such as Ala (**2**), Val (**3**), Leu (**4**), and Ile (**5**) are adsorbed on the AuNPs’ surface through a methyl group. In addition, in these amino acids, the carboxylic group assists them in interacting with the surface of AuNPs. Phe (**6**), Trp (**7**), and Tyr (**13**), which are amino acids with aromatic rings and are generally hydrophobic, based on the calculations, are adsorbed similarly with the aromatic ring in parallel orientation to the gold surface. In the case of Tyr (**13**), the hydroxyl group in the phenyl is oriented toward the gold surface. Met (**8**) adsorption on the AuNPs surface is mediated by the S–CH_3_ group. Pro (**9**) interacts with AuNPs surface through the amine (Au–N) and carboxylic group (Au–O and Au–H–O). Cys (**10**) has a sulfur atom that can covalently bond to the Au atoms on the AuNPs’ surface. Gln (**11**) and Asn (**12**) adsorptions on the surface of the AuNPs take place through the amino group in the side chain. In the case of Ser (**14**) and Thr (**15**), their uptake on the surface of AuNPs occurs via the interaction of the OH group in their side chain through Au–O interaction. Glu (**17**) and Asp (**18**) interact with the surface of AuNPs via the carboxyl group in their side chain, which keeps amino acids close to the surface. The amino acids having a NH_2_ functional group with a positive charge, His (**19**), Lys (**20**), and Arg (**21**), are adsorbed from the amine group on the surface of AuNPs by the Au–N interaction.

**Scheme 1:**
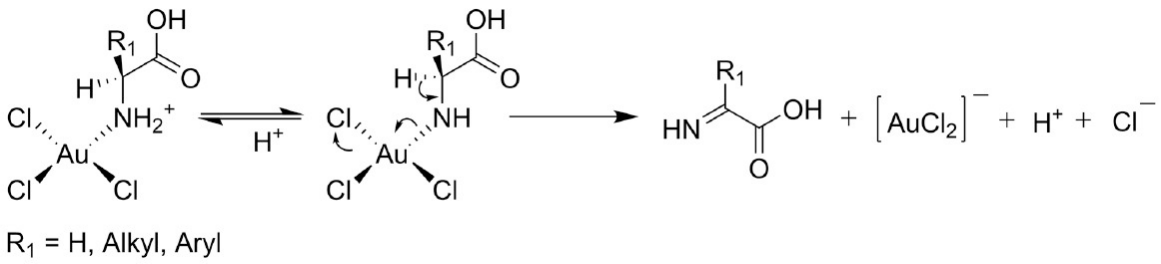
Proposed Mechanism of Rate-Determining Step of the AuCl_4_^–^ Reduction with α-Amino Acids, Which Is Based on the Mechanism Proposed by Zou for l-Glycine^31^

Previous studies have also reported the reduction capability of α-AA toward chloroauric acid.^31−57^ However, as mentioned in the Introduction, little discussion has been dedicated to the mechanism of gold salts reduction with α-amino acids.^31,34,38,43^ For example, Zou et al. proposed a mechanism of glycine oxidation by AuCl_4_^–^ in which in the rate-determining step, gold ions are reduced to the AuCl_2_^–^ ion and l-glycine is reduced to imine, which then hydrolyzes to glyoxylic acid (Scheme 1).^31^ The same reaction pathway was also proposed for the oxidation of l-phenylalanine and l-tryptophane.^52^

**Scheme 2:**
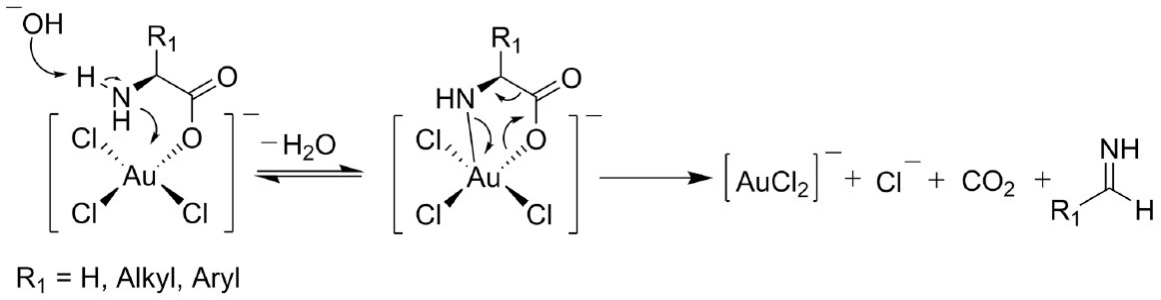
Proposed Mechanism of Rate-Determining Step of the AuCl_4_^–^ Reduction with α-Amino Acids Anions, Which Is Based on the Mechanism Proposed by Ojea-Jiménez for Citrate^60,61^

In our previous studies,^20^ we showed that the mechanism of the rate-determining step of gold salts reduction with α-hydroxycarboxylates (citrate and nine other compounds) follows the mechanism proposed by Ojea-Jiménez et al.^60,61^ Taking into consideration the structural similarities of α-amino acids and α-hydroxy acids, the position of −NH_2_ and −OH to the carboxyl group, we proposed here a similar mechanism of the rate-determining step for salts of α-amino acids (Scheme 2). The crucial differences between mechanisms shown in Schemes 1 and 2 are amino acid oxidation products. As we discussed later, observations made during our studies indicate that the oxidation of amino acids at the applied experimental conditions occurs via the mechanism shown in Scheme 2.

In the proposed mechanism, a ligand exchange reaction of the AuCl_4_^–^ ion with α-amino acid anions leads to an intermediate complex, which upon ring closure forms a cyclic five-membered complex. In the rate-determining step of the reaction, the concerted decarboxylation of cyclic intermediate and the reduction of Au(III) species occur. The resulting Au(I) ions undergo then disproportionation to form Au(0) atoms. The critical difference between α-hydroxycarboxylates and α-amino acid anions is that in the former case acetonedicarboxylate is formed, which accelerates the autocatalytic growth of the seed particles.^58^ In the case of α-amino acid anions, imines are formed due to intermediate complex decomposition. How they affect further steps of AuNPs formation requires additional investigation. However, because imines are unstable and prone to hydrolysis in the presence of water, they quickly convert to corresponding aldehydes in used reaction conditions, which was proven by observations made during experiments. Such formed aldehydes can also reduce gold ions and influence the results of AuNPs synthesis with a particular α-amino acid. The mechanism shown in Scheme 2 should apply to all α-amino acid anions; however, as we discuss below, the mechanism of the rate-determining step is dependent on their molecular structure. Based on the results of our studies described below, the mechanism proposed in Scheme 2 is undoubtedly valid for at least 11 α-amino acids, including α-amino acids with an aliphatic (**1**–**5**), an aromatic (**6**), an amide (**12**), a hydroxyl, and an acidic (**17**, **18**) side chain. For all other α-amino acids, this mechanism can compete, be disturbed, or be replaced with other possible reaction pathways.

**Figure 1:**
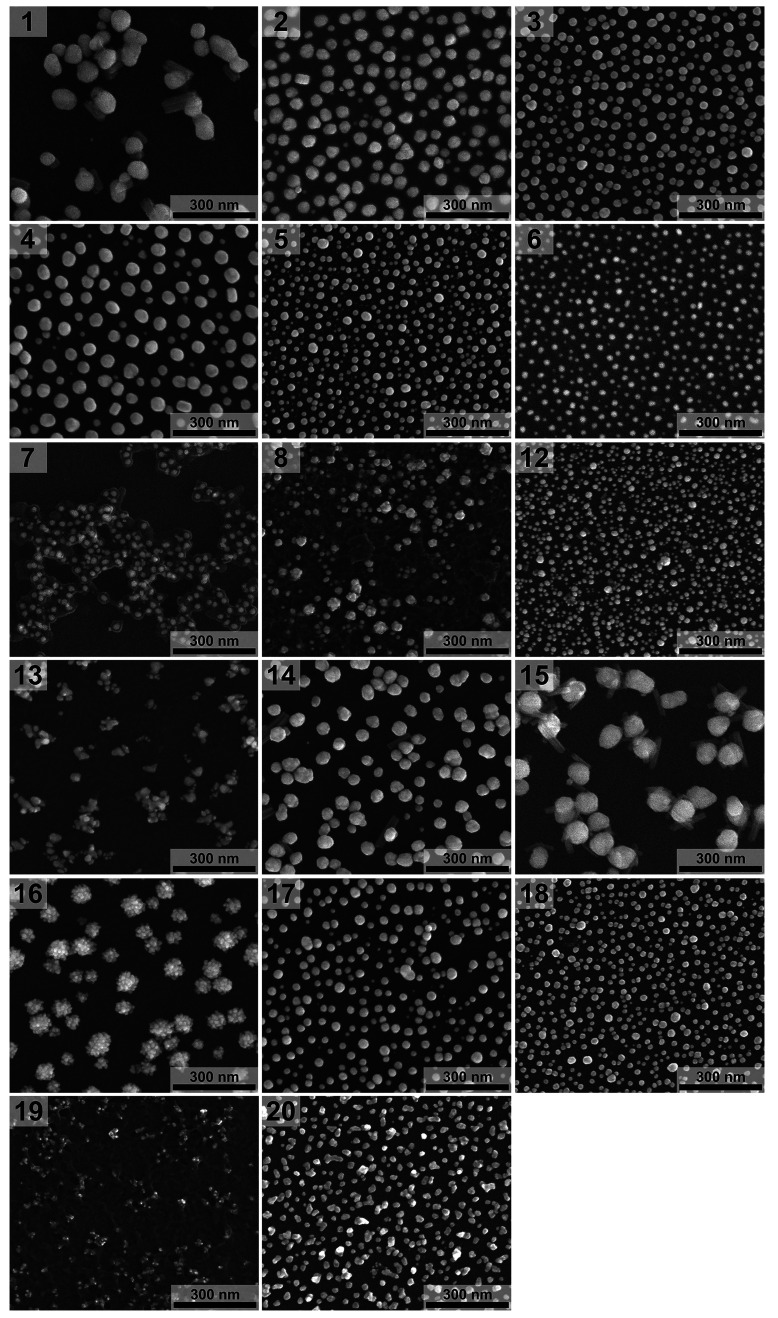
SEM images of AuNPs synthesized using amino acids **1**–**8** and **12**–**20**.

**Figure 2:**
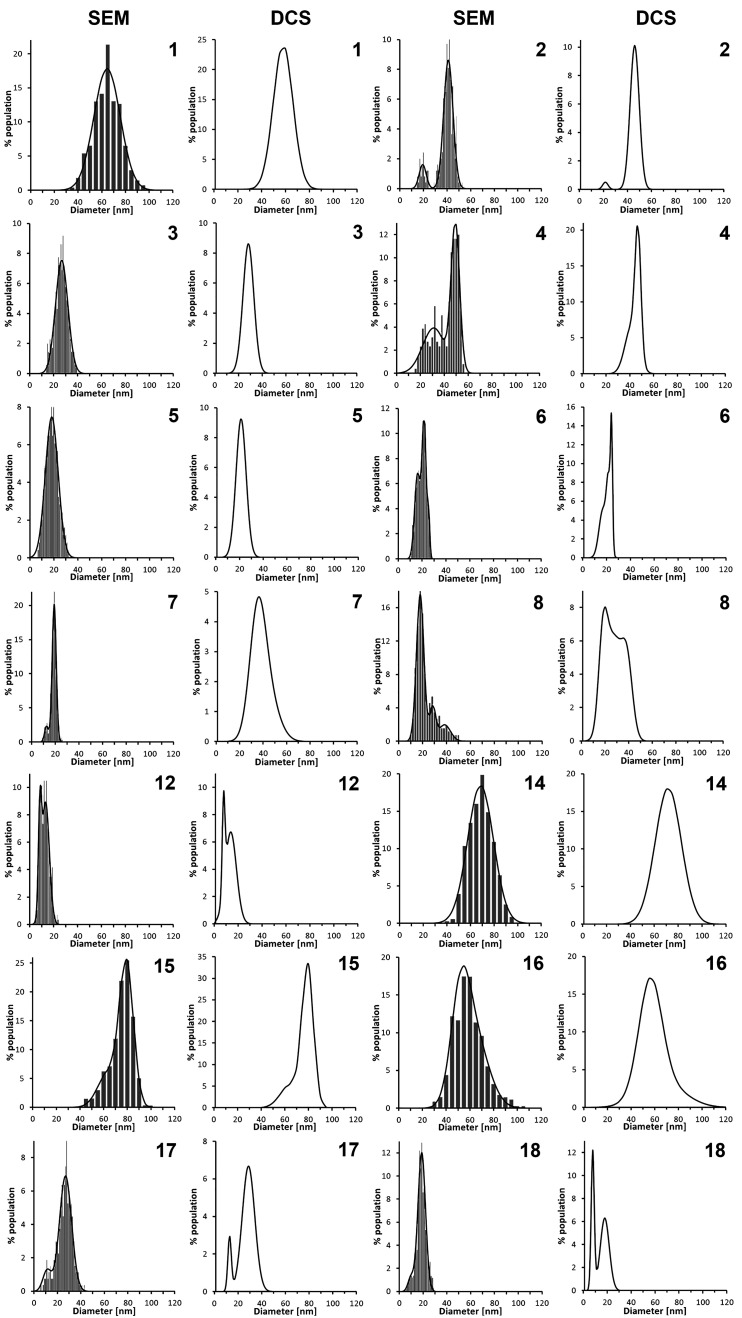
Size distributions of AuNPs synthesized using amino acids **1**–**8**, **12**, and **14**–**18** determined by SEM images analysis and number-weighted size distribution obtained by DCS.

SEM images of AuNPs synthesized with anions of α-amino acids **1**–**8** and **12**–**20** are presented in Figures 1 and S1 (enlarged images). Because of a lack of reduction or aggregation, we did not analyze by SEM reactions’ products of α-AA **9**–**11** and **21**. The size distributions determined based on the SEM images analysis and number-weighted size distributions determined by DCS are shown in Figures 2 and S2. These data were provided only for AuNPs synthesized with salts of α-AA **1**–**8**, **12**, and **14**–**18**, for which such analysis was possible. However, in the case of AuNPs made with salts of α-AA **8** and **16**, it has to be mentioned that they are in the shape of nanoclusters and not quasi-spherical particles. Therefore, we provide their estimated sizes and size distributions. We used in our studies DCS technique because it has been shown to provide sizes and resolutions similar to the TEM technique while providing better statistics due to the analysis of a larger number of particles in a shorter time than TEM.^20,65^ The size distributions determined based on SEM and DCS analysis results, the reaction times for each reducing agent, and the zeta potential ζ and λ_max_ of the resulting AuNPs (for which specific analyses were possible) are compared in Table 1.

**Table 1:** Parameters of Size Distribution (Mean Diameter *d*, Standard Deviation SD, and Relative Standard Deviation RSD), Zeta Potential ζ, and Wavelength of Maximum Absorbance λ_max_ of AuNPs Obtained Using Reducing Agents **1**–**8** and **12**–**20**^a^

		SEM measurements			DCS measurements				
reducing agent	reaction duration [min]	*d* [nm]	SD [nm]	RSD [%]	*d* [nm]	SD [nm]	RSD [%]	ζ [mV]	λ_max_ [nm]
**1**	10	65	11	17	58	8	14		542
**2**	10	41	4	10	46	4	9	36 ± 6	528
		19	3	16	22	2	9		
**3**	9	27	5	19	28	5	18	31 ± 1	522
**4**	10	49	4	8	47	3	6	24 ± 1	526
		31	9	29	42	6	14		
**5**	9	18	5	28	21	4	19	28 ± 1	518
**6**	8	26	1	4	24	1	4	31 ± 1	524
		22	2	9	22	2	9		
		16	3	19	17	3	18		
**7**	5	19	2	10	35	7	20		552
		13	2	15					
**8**	11	38	5	13	39	5	13		540
		29	3	10	27	7	26		
		18	3	17	18	4	22		
**12**	7	13	3	23	14	5	36	33 ± 2	522
		8.5	1.3	15	7.8	1.2	15		
**13**	5								554
**14**	13	69	11	16	72	11	15	37 ± 3	534
**15**	20	79	6	8	79	5	6		562
		64	9	14	65	9	14		
**16**	9	64	12	19	67	19	28		616
		52	8	15	56	10	18		
**17**	11	27	5	19	29	5	17	42 ± 1	520
		11	4	36	12	1	8		
**18**	8	19	3	16	18	4	22	34 ± 2	522
		10	3	30	7.9	1.3	16		
**19**	30								546
**20**	11								554

a The reaction time is provided for each reducing agent.

In the reaction of salts of α-amino acids **1**–**21** with chloroauric acid, the reduction of gold ions indicated by the changes of the reaction mixture color over time was observed for salts of all studied α-amino acids except l-cysteine (**10**). In the case of **10**, the reaction resulted in the formation of the yellowish precipitate, likely elemental sulfur, being a product of a decomposition of cystine formed from the redox reaction of **10**.^41,66^ Interestingly, in another study formation of black precipitate was observed in a reaction of **10** with tetrachloroaurate, which was associated with possible complexation of cysteine to the tetrachloroaurate salt via a sulfur atom.^56^ In the case of three α-amino acids, l-proline (**9**), l-glutamine (**11**), and l-arginine (**21**), the reduction of gold salts was observed; however, it was followed by quick aggregation of formed reaction products. Our results for these three α-AA are consistent with those obtained in other studies; however, the authors did not explain observed reactions (Table S1).^56,57^ The possible explanation of obtained results can be associated with several factors, including metal ion complexation (known to be strong for l-cysteine, l-histidine, and l-methionine), the metal-binding affinity of amino acids to the metal surface,^55^ and structure of intermediate decomposition (Scheme 2), which may or may not facilitate the further formation of AuNPs.^19,20,58−61^

The reduction of HAuCl_4_ with salts of 11 studied α-amino acids, **1**–**6**, **12**, **14**, **15**, **17**, and **18**, which constitute α-amino acids with aliphatic (Gly (**1**), Ala (**2**), Val (**3**), Leu (**4**), and Ile (**5**)), aromatic (Phe (**6**)), amidated (Asn (**12**)), hydroxylated (Ser (**14**) and Thr (**15**)), and acidic (Glu (**17**) and Asp (**18**)) side chains yielded mono- or polydisperse quasi-spherical AuNPs with sizes ranging from 10 to over 60 nm and different size distributions (Figures 1, 2, S1, and S2; Table 1). Considering the molecular structure of these 11 α-AA (no functional groups on side chains capable of reducing tetrachloroaurate ions) and the results of their reactions with HAuCl_4_, it can be confirmed that the rate-determining step of gold salts reduction with α-AA anions occurs via mechanism proposed in Scheme 2. This proposal is additionally proved by the observations made during the reaction of dl-phenylalanine (**6**), in which the smell of flowers was perceptible. This smell is likely associated with the initial formation of 2-phenylethanimine followed by its hydrolysis to phenylacetaldehyde, a compound added to fragrances to impart hyacinth, narcissi, or rose nuance. Similarly, during the synthesis of AuNPs with a salt of l-isoleucine (**5**), an unpleasant odor was perceptible, which can be associated with the initial formation of imine, which hydrolyzes to 2-methylbutyraldehyde.

In the case of α-amino acids **2**–**6**, **12**, **17**, and **18**, AuNPs were in the form of monodisperse (**3** and **5**) or polydisperse (**2**, **4**–**6**, **12**, **17**, and **18**) quasi-spherical nanoparticles with sizes ranging from 10 to 50 nm (Figures 1, 2, and S1; Table 1). The polydispersity of synthesized AuNPs can be related to the reactivity of the oxidation products of α-amino acids, either imines or aldehydes formed in imine hydrolysis. Such newly formed compounds can reduce gold ions via a different mechanism than α-amino acids, forming new families of AuNPs. Interesting observation, which was also previously reported,^56^ are different results of gold salts reduction with l-glutamine (**11**) and l-asparagine (**12**), having the same amide group at the side chain but differing by one −CH_2_– group. Both α-AA are capable of HAuCl_4_ reduction, but only in reaction with **12** stable AuNPs are formed. The AuNPs made with α-AA **1**, **14**, and **15** are quasi-spherical, and their sizes are over 60 nm (Figures 1, 2, S1, and S2; Table 1). The AuNPs synthesized with these three α-AA have relatively broad monomodal (**1** and **14**) or multimodal size distributions (**15**). The AuNPs synthesized with all 11 α-amino acids (**1**–**6**, **12**, **14**, **15**, **17**, and **18**) except l-glycine (**1**) are stable over time (Figure S3). The AuNPs synthesized with l-glycine (**1**) aggregate after 1 day, which is likely associated with a small molecular size of **1**, which does not allow the formation of a good stabilizing barrier on the AuNPs surfaces, and also a relatively large size of AuNPs that Gly molecules should stabilize. Our results for these 11 α-AA differ from those obtained in previously reported studies; however, this is not surprising because various reaction conditions have been used (Table S1).^31−57^ In addition, in three studies in which all 20 proteinogenic α-amino acids were used, only limited information is provided regarding the results of synthesized AuNPs characterization.^55−57^ However, based on the available information, we can conclude that the synthesis conditions used in our studies provided better results than other relevant studies (Table S3).^55−57^

The syntheses, with the use of α-amino acids **7**, **8**, and **19**, resulted in the formation of either quasi-spherical (l-tryptophan (**7**)) or irregular (l-methionine (Met) (**8**) and l-histidine (His) (**19**)) AuNPs with sizes in the range of 10–20 nm for **7**, 10–50 nm for **8**, and ca. 10 nm for **19**. A common feature of all these nanoparticles is the presence of organic matter surrounding AuNPs (**7**) or in which AuNPs are embedded (**8** and **19**). Our results for l-tryptophan (**7**) agree with the results reported by Selvakannan et al.^34^ However, due to different concentrations of reagents and higher reaction temperatures, we have obtained AuNPs with smaller sizes (Table 1). The organic shell capping and stabilizing AuNPs synthesized with **7**, containing imidazole in the side chain, is most likely made of polytryptophan resulting from oxidation of the α-amino acid.^34^ This organic shell is responsible for differences in AuNPs sizes and size distributions obtained from SEM and DCS measurements. Similar processes likely occur for l-histidine (**19**), containing imidazole in the side chain, and the organic matter observed in SEM images (Figures 1 and S1) is made of polyhistidine. In the case of l-methionine (**8**), the formation of organic matter/film in which AuNPs were embedded was also observed by Laban et al.^38^ This organic matter was assumed to be a layer of amino acid adsorbed on the AuNPs surface upon reduction and during Au nucleation and aggregation processes. Our studies also showed that gas with a distinctively putrid smell was produced during the reaction, likely methanethiol. This observation indicates that **8** undergoes decomposition in applied reaction conditions; therefore, the formation of the organic matter observed on SEM images can also be associated with this process. Laban and coauthors have also proposed the mechanism of gold ions reduction by **8**, in which, in the first and fastest step, the formation of [Au^3+^Cl_3_(l-methionine)]^−^ occurs, which results from a nucleophilic attack of S donor from the thioether group and Cl^–^ substitution. In the next slow step, the second l-methionine molecule promotes Au^3+^ reduction by forming a linear two-coordinated Au^+^–l-methionine complex and further disproportioning aurous species to gold atoms with the formation of methionine sulfoxide.^38^ The formation of AuNPs in reactions of HAuCl_4_ with Met can be governed by mechanisms proposed by Laban and by us, which could explain such a broad AuNPs size distribution (Figure 2).

The syntheses, with the use of three remaining α-amino acids, l-tyrosine (**13**), l-(4)-hydroxyproline (**16**), and l-lysine (**20**), resulted in the formation of irregular AuNPs with sizes in the range of 20–60 nm (**13** and **20**) and AuNPs in the form of nanoclusters with sizes in the range of 25–95 nm (**16**) (Figures 1, 2, and S1; Table 1). The reactivity of l-tyrosine (**13**) toward tetrachloroaurate ions was proposed to be governed by the presence of a cresol component in the side chain, which is known to be easily oxidized into the quinone in the air.^67,68^ According to the previous reports, l-tyrosine reduces gold ions to form nanoparticles using its phenolic group under alkaline conditions where it is oxidized into a semiquinone group, and the amine and carboxylic acid groups remain the same after the formation of nanoparticles.^43,50^ Interestingly, despite similar reaction conditions, our results differ from previously reported studies,^43,50^ spherical vs irregular AuNPs, which are likely associated with differences in the order of added reagents and concentrations. The reversing of the order of addition in the Turkevich method has been shown previously to influence the results of gold ions reduction with citrate.^69,70^ The results observed for l-lysine (**20**) differ from previously reported,^54^ again likely due to different reaction conditions. Reactions were performed at room temperature and in the dark, and different concentrations of reagents were used. The reaction likely occurs in the rate-determining step via the mechanism proposed in Scheme 2; however, the final formation of irregular AuNPs may be associated with an amine group in the side chain, which can bind to the surface of formed AuNPs.

According to our knowledge, l-(4)-hydroxyproline (**16**), the only non-proteinogenic α-amino acid in our studies, was used as a reducing and capping agent in AuNPs for the first time. The observed results of its reaction with HAuCl_4_ are very interesting, especially considering results obtained for l-proline (**9**), which reduced gold ions but formed reaction products aggregated. Therefore, it can be assumed that observed differences in the reactivities of **9** and **16** toward tetrachloroaurate ions are associated with introducing the hydroxyl group into the pyrrolidine ring. However, the mechanism for the reaction of **16** toward HAuCl_4_ has to be further elucidated.

### Optical Properties of Synthesized AuNPs

**Figure 3:**
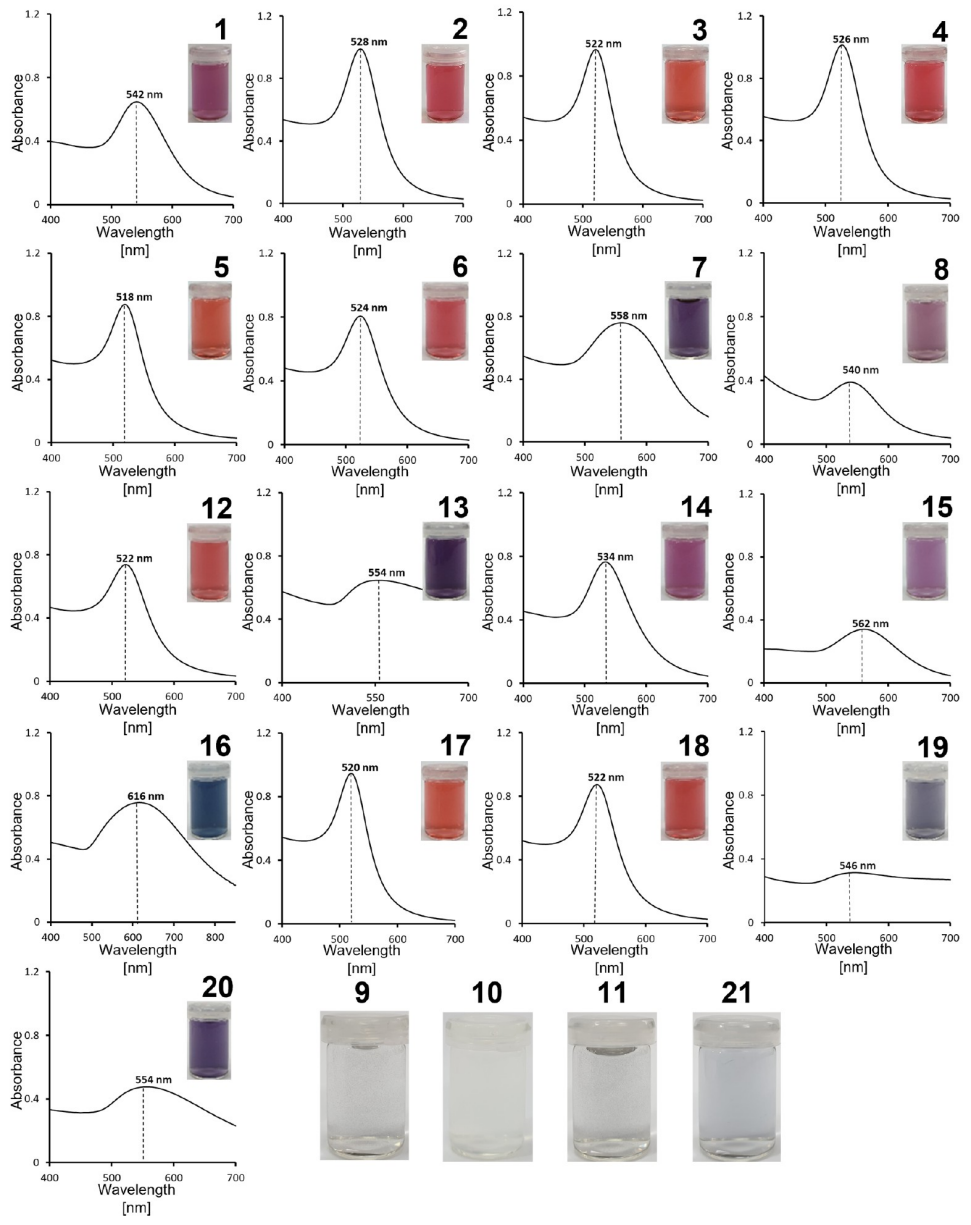
Absorption spectra of AuNPs synthesized with salts of amino acids **1**–**8** and **12**–**20** with indicated λ_max_. Colors of AuNPs samples on white background are shown in the inset. In addition, solutions resulting from HAuCl_4_ reduction using α-AA **9**–**11** and **21** are shown for comparison.

The absorption spectra of synthesized AuNPs in the range 400–700 nm and images of their water suspensions are shown in Figure 3. The suspensions of AuNPs synthesized with amino acids **2**–**6**, **12**, **17**, and **18** were red, which is consistent with their narrow absorption bands with similar intensities and λ_max_ falling in a range of 518–528 nm.^71^ For all these amino acids, AuNPs were mono- or polydisperse and quasi-spherical with sizes ranging from 10 to 50 nm (Figures 1, 2, and S1; Table 1). The suspensions of AuNPs synthesized with amino acids **1**, **14**, and **15** were violet. The AuNPs made with these amino acids are quasi-spherical, but their sizes are over 60 nm, and they have relatively broad, mono- or multimodal size distributions (**1** and **14**, **15**). An increasing red-shift of the absorption maxima was observed with the increasing size of AuNPs, and the absorption bands were broader than for AuNPs made with amino acids **2**–**6**, **12**, **17**, and **18**. In the case of AuNPs synthesized with amino acids **7** and **8**, their optical properties are associated with three factors: the shape of particles, their dielectric environment, and agglomeration state. The product of the reaction of **7** with HAuCl_4_ undergoes polymerization with the formation of an organic shell surrounding quasi-spherical AuNPs, which changes the local dielectric environment but also causes agglomeration (Figure 1). The optical properties of AuNPs synthesized from **8** are affected by their shape and presence of organic matter, which resulted from the reduction of gold salts with **8**. Similar observations can be made for AuNPs synthesized with amino acid **19**, although much smaller NPs are formed. In the case of samples **13** and **20**, where AuNPs of multiple shapes are present, the absorption band is much broader and has a lower intensity than for other samples. This effect arises from a combination of the plasmon resonances of families of particles with various shapes and sizes. The optical properties of AuNPs synthesized with amino acid **16** are associated with the shape of nanoclusters. These AuNPs have a broad plasmon resonance band, with λ_max_ having the largest red-shift (616 nm) among all synthesized AuNPs. In the case of reactions with amino acids **9**–**11** and **21**, either aggregation of just formed nanostructures or no reduction was observed. Therefore, the final solutions were colorless.

## Conclusions

In summary, it has been shown that the simple green reagents, α-amino acids, can reduce gold ions and cap/stabilize such synthesized gold nanoparticles. By applying the standard Turkevich method conditions for most studied amino acids, AuNPs with mean diameters from several to tens of nanometers and good stability can be reproducibly synthesized simply by a change of reducing agent. Out of the twenty-one investigated 12 α-amino acids, **1**–**7**, **12**, **14**, **15**, **17**, and **18** yielded in the synthesis quasi-spherical gold nanoparticles with either unimodal or multimodal size distributions. α-Amino acids **8**, **13**, **19**, and **20** reduced gold ions to form AuNPs; however, the resulting nanoparticles were of irregular shapes. The only investigated non-proteinogenic α-amino acid, l-(4)-hydroxyproline (**16**), yielded in reaction with HAuCl_4_ gold nanoparticles in the form of nanoclusters. Among the twenty-one investigated amino acids, only four, l-proline (**9**), l-cysteine (**10**), l-glutamine (**11**), and l-arginine (**21**), have not yielded gold nanoparticles, although all of them but **9** reduced gold salts. Based on the results of our studies, the mechanism proposed in Scheme 2, in which a cyclic five-membered complex is formed, is undoubtedly valid for at least 11 α-amino acids, including Gly (**1**), Ala (**2**), Val (**3**), Leu (**4**), Ile (**5**), Phe (**6**), Asn (**12**), Ser (**14**), Thr (**15**), Glu (**17**), and Asp (**18**)). For all other α-amino acids, this mechanism can compete, be replaced, or be disturbed with other possible reaction pathways.

## Experimental Section

### Chemicals

l-Alanine (C_3_H_7_NO_2_, 99%), l-valine (C_5_H_11_NO_2_, 99%), l-isoleucine (C_6_H_13_NO_2_, 99%), dl-phenylalanine (C_9_H_11_NO_2_, 99%), l-tryptophan (C_11_H_12_N_2_O_2_, 99%), l-proline (C_5_H_9_NO_2_, 99%), l-glutamine (C_5_H_10_N_2_O_3_, 99%), l-asparagine (C_4_H_8_N_2_O_3_, 99%), l-tyrosine (C_9_H_11_NO_3_, 99%), l-serine (C_3_H_7_NO_3_, 99%), dl-threonine (C_4_H_9_NO_3_, 99.5%), l-glutamic acid (C_5_H_9_NO_4_, 99%), l-aspartic acid (C_4_H_7_NO_4_, 98+%), and l-histidine (C_6_H_9_N_3_O_2_, 98%) were purchased from Acros Organics. l-Glycine (C_2_H_5_NO_2_, 98.5% p.a.) was purchased from POCh S.A. l-Leucine (C_6_H_13_NO_2_, 99%) and l-arginine (C_6_H_14_N_4_O_2_, 98%) were purchased from Alfa Aesar.

l-(4)-Hydroxyproline (C_5_H_9_NO_3_, 99%) was purchased from Riedel-de Haën. l-Methionine (C_5_H_11_NO_2_S, ≥99.5%) was purchased from Sigma-Aldrich. l-Cysteine (C_3_H_7_NO_2_S) was purchased from AppliChem GmbH. l-Lysine (C_6_H_14_N_2_O_2_, ≥97%) was purchased from SAFC. Sodium hydroxide (NaOH, 98.8%) was purchased from POCH Basic, and gold(III) chloride hydrate (HAuCl_4_·H_2_O, 30% Au) was purchased from Sigma-Aldrich. The purchased amino acids were converted to corresponding salts by reacting them with sodium hydroxide in an amount corresponding to the acid moles number multiplied by the number of carboxy groups in the molecule. The calculated amount of sodium hydroxide was used with a 10% excess. Reacting of some amino acids with NaOH was not sufficient to dissolve them, and therefore in the case of l-glutamic acid, l-aspartic acid, l-asparagine, l-serine, dl-threonine, l-(4)-hydroxyproline, and l-tyrosine, it was necessary to heat their solutions. Structures of all amino acids used in the studies described here are shown in Chart 1. Ultrapure deionized (DI) water (18.2 MΩ·cm at 25 °C, Hydrolab, Poland) was used throughout the experiments. All glassware was treated before the reactions with aqua regia for 5 min and rinsed several times with DI water.

### Synthesis of Gold Nanoparticles

The solutions of AuNPs were prepared following the method described by Turkevich and successfully used in AuNPs synthesis with different α-hydroxy acids.^19,20^ In a typical procedure, 3 mL of HAuCl_4_ aqueous solution (5 mM) was added to a 250 mL flask containing 54 mL of water. The solution was brought to a boil while stirring magnetically (400 rpm), and then 3 mL of α-amino acid salt aqueous solution (20 mM) was added at once. The reaction was performed until the solution acquired a color (red, purple, or blue) that remained unchanged for another 5 min.

### Characterization of Gold Nanoparticles

The morphology and size distribution of synthesized nanoparticles were analyzed using scanning electron microscopy (SEM, Quanta 3D FEG, FEI) at an accelerating voltage of 20 kV. The samples for SEM analysis were prepared following the procedure described in the NIST protocol.^72^ The amine-functionalized Si chips made from silicon wafer were placed in Eppendorf tubes, and solutions of synthesized AuNPs were added. The tubes were shaken for 24 h at 400 rpm. Each sample was then rinsed with deionized water and dried in air at room temperature. Statistical analysis was performed on the SEM images using the Digimizer software. At least 200 particles were measured to assess their mean size and size distribution. Nanoparticle size distributions were additionally analyzed by the DCS technique using a CPS Disc Centrifuge MOD DC24000 UHR (CPS Instruments Inc.).^20,73^ The analyzed samples were injected for sedimentation into a centrifugation disc filled with gradient fluid (DI water solution of sucrose with a density gradient–the concentration of sucrose varied from 4 to 12% w/w), spinning at 22000 rpm. The accuracy of the measured size was ensured by calibration performed before each measurement using silica particles (0.145 μm, CPS Instruments Inc.) as a calibration standard. The size distributions were obtained from 100 μL of freshly synthesized colloidal solution of AuNPs. Matlab software was used to approximate the results obtained from SEM and DCS measurements with normal distribution and calculate the mean value and standard deviation.

Zeta potential measurements were performed by phase analysis light scattering (PALS) technique using a NanoBrook Omni (Brookhaven Instruments) instrument with a 640 nm diode laser. In a typical experiment, a colloidal solution of AuNPs was filtered three times using syringe filters (Supor Membrane, pores 0.22/0.45/1.2 μm, ϕ 25 mm, Pall Corporation; hydrophilic filters, pores 0.22/0.45/0.8 μm, ϕ 25 mm, ChemLand; nylon66, pores 0.22 μm, ϕ 25 mm, ChemLand). 1.5 mL of the solution was placed into a standard disposable plastic cuvette, and then a two-electrode arrangement was introduced into the cuvette. The measurements were performed at room temperature (25 °C). The laser power and voltage of the electrodes were adjusted automatically. The zeta potential was assessed based on three measurements of 40 cycles each (RMS residual below 0.1) and calculated using the Smoluchowski equation.

The absorption spectra of AuNPs colloidal solutions were measured at room temperature using a Lambda 650 UV–vis spectrophotometer (PerkinElmer) in the 400–700 nm spectral range. Spectra of freshly prepared AuNPs colloidal solutions were measured in a quartz cuvette (1 cm optical path) placed inside the integration sphere, which allowed to measure of pure absorbance of synthesized AuNPs.

## Supporting Information Available

Tables with comparison of obtained results with results of other relevant studies, table with amino acids properties, enlarged SEM images, results of SEM and DCS analysis, and data regarding AuNPs stabilities (PDF)

## Abbreviations

AuNPs: gold nanoparticles
NPs: nanoparticles
α-AA: α-amino Acids
DCS: differential centrifugal sedimentation
PALS: phase analysis light scattering
SEM: scanning electron microscopy
SERS: surfaced-enhanced Raman spectroscopy.
