# Supplementary material for: α-Amino Acids as Reducing and Capping Agents in Gold Nanoparticles Synthesis Using the Turkevich Method
Source: Langmuir. 2023 Jun 14;39(25):8646–57. doi: 10.1021/acs.langmuir.3c00507 (PMC10308821; doi:10.1021/acs.langmuir.3c00507)
Supplement: Supplementary file 1 — la3c00507_si_001.pdf [file la3c00507_si_001.pdf]

Supporting Information

α-Amino Acids as Reducing and Capping Agents in Gold Nanoparticles Synthesis Using Turkevich Method

Aleksandra M. Figat,[a] Bartosz Bartosewicz,[a] Malwina Liszewska,[a] Bogusław Budner,[a] Małgorzata Norek,[b] Bartłomiej J. Jankiewicz[*,a]*

[a]Institute of Optoelectronics, Military University of Technology, gen. Sylwestra Kaliskiego 2, 00-908 Warsaw, Poland

[b]Faculty of Advanced Technologies and Chemistry, Military University of Technology, gen. Sylwestra Kaliskiego 2, 00-908 Warsaw, Poland

***Corresponding Author**

Bartłomiej J. Jankiewicz

Institute of Optoelectronics, Military University of Technology, gen. Sylwestra Kaliskiego 2, 00-908 Warsaw, Poland

E-mail: bartlomiej.jankiewicz@wat.edu.pl; Phone: +48 261 837 639; Fax: +48 22 666 895

Tables S1. Comparison of obtained results with results of other relevant studies.[1-26]

#	AA	Ref.	Reaction Conditions			Reaction products		Comments
			**Au source**	**Additional reagents**	**Concentration of AA; Temperature; Reaction Time; pH**	**AuNPs (size [nm]; shape)**	**AuNPs Our studies**	
		**Our article**	5 mM $HAuCl_4$	NaOH	20mM AA; boiling; 5-30 min			
**1**	**Gly**	1	0.1 mM $HAuCl_4$	No	0.7 mM AA; R.T.; 30-36 h	N.A.	64.5 nm (SEM) spherical	The authors stated that a pinkish-red AuNPs solution was formed, but no SEM or UV-vis spectra were provided. Reactions were carried out in µL-scale and, in some cases, with the addition of DMSO.
		2	10 mL 0.01% (w/v) $HAuCl_4$	No	10 mL 1.0 mM AA; boiling	No reduction		The authors used 0.1-5.0 mM concentrations of AA. We used for comparison results of syntheses for AA concentration 1.0 mM, for which the largest number of used AA gave stable nanoparticles. There is no complete information on AuNPs characterization results.
		3	0.5 mM $HAuCl_4$	1 M NaOH or 1 M HCl	50 mM AA; 80 °C; pH 7.1–7.8	N.A. but stable colloids formed		The characterization of optical properties, size, and size distributions (DLS, TEM) were done only for His.
		4	$HAuCl_4$ (Au^{3+} 0.05 mg/mL)	0.5 mol/L NaOH	AA:Au^{3+} 10:1 (w/w); R.T.; 0.5 h in dark then 4 h under UV (365 nm); pH 10	39 ± 8 nm (TEM) 47 ± 1 nm (DLS) spherical		After irradiation, $HAuCl_4$/Gly solution changed its color to purple-red.
**2**	**Ala**	1	0.1 mM $HAuCl_4$	No	0.7 mM AA; R.T.; 30-36 h	N.A.	41.1 nm and 19.4 nm (SEM) spherical	The authors stated that a pinkish-red AuNPs solution was formed, but no SEM or UV-vis spectra were provided. Reactions were carried out in µL-scale

								and, in some cases, with the addition of DMSO.
		2	10 mL 0.01% (w/v) $HAuCl_4$	No	10 mL 1.0 mM AA; boiling	No reduction		The authors used 0.1-5.0 mM concentrations of AA. We used for comparison results of syntheses for AA concentration 1.0 mM, for which the largest number of used AA gave stable nanoparticles. There is no complete information on AuNPs characterization results.
		3	0.5 mM $HAuCl_4$	1 M NaOH or 1 M HCl	50 mM AA; 80 °C; pH 7.1–7.8	N.A. but stable colloids formed		The characterization of optical properties, size, and size distributions (DLS, TEM) were done only for His.
**3**	**Val**	1	0.1 mM $HAuCl_4$	No	0.7 mM AA; R.T.; 36-48 h	N.A.	26.7 nm (SEM) spherical	The authors stated that a pinkish-red AuNPs solution was formed, but no SEM or UV-vis spectra were provided. Reactions were carried out in μL-scale and, in some cases, with the addition of DMSO.
		2	10 mL 0.01% (w/v) $HAuCl_4$	No	10 mL 1.0 mM AA; boiling	~75 nm (DLS) spherical		The authors used 0.1-5.0 mM concentrations of AA. We used for comparison results of syntheses for AA concentration 1.0 mM, for which the largest number of used AA gave stable nanoparticles. There is no complete information on AuNPs characterization results.
		3	0.5 mM $HAuCl_4$	1 M NaOH or 1 M HCl	50 mM AA; 80 °C; pH 7.1–7.8	N.A. but stable colloids formed		The characterization of optical properties, size and size distributions (DLS, TEM) were done only for His.
**4**	**Leu**	1	0.1 mM $HAuCl_4$	No	0.7 mM AA; R.T.; 36-48 h	N.A.	49.3 nm and 31.1 nm (SEM) spherical	The authors stated that a pinkish-red AuNPs solution was formed, but no SEM or UV-vis spectra were provided. Reactions were carried out in μL-scale

								and, in some cases, with the addition of DMSO.
		2	10 mL 0.01% (w/v) $HAuCl_4$	No	10 mL 1.0 mM AA; boiling	~70 nm (DLS) spherical		The authors used 0.1-5.0 mM concentrations of AA. We used for comparison results of syntheses for AA concentration 1.0 mM, for which the largest number of used AA gave stable nanoparticles. There is no complete information on AuNPs characterization results.
		3	0.5 mM $HAuCl_4$	1 M NaOH or 1 M HCl	25 mM AA; 80 °C; pH 7.1–7.8	Aggregation		The characterization of optical properties, size, and size distributions (DLS, TEM) were done only for His.
		4	$HAuCl_4$ (Au^{3+} 0.05 mg/mL)	0.5 mol/L NaOH	AA:Au^{3+} 10:1 (w/w); R.T.; 0.5 h in dark then 4 h under UV (365 nm); pH 10	25 ± 7 nm (TEM) 27 ± 2 nm (DLS) spherical		After irradiation, $HAuCl_4$/Leu solution changed its color to rose-red.
		5	100 mL 0.254 mM $HAuCl_4$	No	10 mL 10.0 mM AA (Au:AA 1:4 molar ratio); boiling; 5 min	62 ± 8 nm (TEM for spherical) Various shapes (spheres, triangles, rodes, pentagons)		Authors used in one method citrate for reduction of $HAuCl_4$ and then displaced citrate ions by amino acid. In the second method they used the amino acid both as reducing and stabilizing agent.
**5**	**Ile**	1	0.1 mM $HAuCl_4$	No	0.7 mM AA; R.T.; 48-72 h	N.A.	18.2 nm (SEM) spherical	The authors stated that a pinkish-red AuNPs solution was formed, but no SEM or UV-vis spectra were provided. Reactions were carried out in µL-scale and, in some cases, with the addition of DMSO.
		2	10 mL 0.01% (w/v) $HAuCl_4$	No	10 mL 1.0 mM AA; boiling;	~70 nm (DLS)		The authors used 0.1-5.0 mM concentrations of AA. We used for comparison results of syntheses for AA

								concentration 1.0 mM, for which the largest number of used AA gave stable nanoparticles. There is no complete information on AuNPs characterization results.
		3	0.5 mM $HAuCl_4$	1 M NaOH or 1 M HCl	25mM AA; 80 °C; pH 7.1–7.8	Aggregation		The characterization of optical properties, size, and size distributions (DLS, TEM) were done only for His.
**6**	**Phe**	1	0.1 mM $HAuCl_4$	No	0.7 mM AA; R.T.; 3-15 h	N.A.	25.5 nm, 21.7 nm, and 16.1 nm (SEM) spherical	The authors stated that a pinkish-red AuNPs solution was formed, but no SEM or UV-vis spectra were provided. Reactions were carried out in µL-scale and, in some cases, with the addition of DMSO.
		2	10 mL 0.01% (w/v) $HAuCl_4$	No	10 mL 1.0 mM AA; boiling;	~20 (TEM) spherical		The authors used 0.1-5.0 mM concentrations of AA. We used for comparison results of syntheses for AA concentration 1.0 mM, for which the largest number of used AA gave stable nanoparticles. There is no complete information on AuNPs characterization results.
		3	0.5 mM $HAuCl_4$	1M NaOH or 1M HCl	25 mM AA; 80 °C; pH 7.1–7.8.	N.A. but stable colloids formed		The characterization of optical properties, size, and size distributions (DLS, TEM) were done only for His.
		6	5 mM $HAuCl_4$	No	25 mM AA; boiling;	10-20 nm (TEM)<br>29 nm (DLS) spherical		Studies were done for different concentrations.
		7	90 mL 0.1 mM $HAuCl_4$	No	10 mL 1.0 mM AA; boiling; 55 min	14.5 nm (TEM) spherical		Authors used in one method $NaBH_4$ for reduction of $HAuCl_4$ and then displaced borohydride ions by amino acid. In the second method they used the amino acid both as reducing and stabilizing agent.

7	Trp	1	0.1 mM $HAuCl_4$	No	0.7 mM AA; R.T.; 0-1 h	N.A.	18.9 nm and 12.5 nm (SEM) spherical	The authors stated that a pinkish-red AuNPs solution was formed, but no SEM or UV-vis spectra were provided. Reactions were carried out in µL-scale and, in some cases, with the addition of DMSO.
		2	10 mL 0.01% (w/v) $HAuCl_4$	No	10 mL 1.0 mM AA; boiling	Aggregation		The authors used 0.1-5.0 mM concentrations of AA. We used for comparison results of syntheses for AA concentration 1.0 mM, for which the largest number of used AA gave stable nanoparticles. There is no complete information on AuNPs characterization results.
		3	0.5 mM $HAuCl_4$	1 M NaOH or 1 M HCl	25 mM AA; 80 °C; pH 7.1–7.8	N.A. but stable colloids formed		The characterization of optical properties, size, and size distributions (DLS, TEM) were done only for His.
		6	5 mM $HAuCl_4$	No	25 mM AA; boiling	20-30 nm (TEM) 41 nm (DLS) spherical		Studies were done for different concentrations.
		8	0.01% $HAuCl_4$	No	0.5 mg/mL AA; 25 °C; 12 h	60 ± 5 nm (TEM) spherical		Short communication with not too many experimental details.
		9	2 mL 5 mM $HAuCl_4$	1 mL KOH	5 mL 10 mM AA; 92 mL water; boiling; reverse order of reagents addition	8.4 nm (TEM) 21 nm (DLS) spherical		Solution of $HAuCl_4$ was added to solution of AA and KOH in water.
		10	90 mL 0.1 mM $HAuCl_4$	No	10 mL 1.0 mM AA; 50 °C	31.2 ± 1.8 nm (TEM) spherical		Authors used in one method $NaBH_4$ for reduction of $HAuCl_4$ and then displaced borohydride ions by amino acid. In the second method they used the amino acid both as reducing and stabilizing agent.

		11	10 mL $HAuCl_4$	No	15 mL AA; boiling	10-25 nm (TEM) spherical		Not clear what concentrations of $HAuCl_4$ and AA were used. PEG 1000 added to stabilize AuNPs.
		12	1 mL 1 mM $HAuCl_4$	No	0.1 mL 4.8 mM AA; 22, 60 and 80 °C	22 °C<br>20-50 nm (TEM) spherical<br>60 °C<br>50 ± 10 nm edge length (TEM) anisotropic<br>80 °C<br>80 ± 15 nm edge length (TEM) anisotropic		Authors found that the temperature has a major influence upon the crystal growth.
		13	50 mL 0.5 mM $HAuCl_4$	No	50 mL 0.5 mM AA; 25, 37 and 50 °C	25 °C<br>41.5 ± 11.2 nm (TEM) spherical<br>37 °C<br>38.1 ± 9.6 nm (TEM) spherical<br>50 °C<br>34.8 ± 9.3 nm (TEM) spherical		Authors evaluated the influences of reaction temperature, foreign metal ions Ag (I), and surfactants of nonionic (polyethylene glycol, PEG),<br>anionic (sodium dodecyl sulfate, SDS), and cationic (cetyltrimethylammonium bromide, CTAB) on AuNPs synthesis.
		14	3.83 mM $HAuCl_4$	No	0.05 M AA; 37 °C then 25 °C; 72 h	7.8 ± 0.3 nm (TEM)<br>8.8 ± 1.0 nm (DLS)<br>spherical		

**8**	**Met**	1	0.1 mM $HAuCl_4$	No	0.7 mM AA; R.T.; 72-336 h	N.A.	38.4 nm, 28.5 nm, and 18.0 nm (SEM) spherical and irregular	The authors stated that a pinkish-red AuNPs solution was formed, but no SEM or UV-vis spectra were provided. Reactions were carried out in µL-scale and, in some cases, with the addition of DMSO.
		2	10 mL 0.01% (w/v) $HAuCl_4$	No	10 mL 1.0 mM AA; boiling	No reduction		The authors used 0.1-5.0 mM concentrations of AA. We used for comparison results of syntheses for AA concentration 1.0 mM, for which the largest number of used AA gave stable nanoparticles. There is no complete information on AuNPs characterization results.
		3	0.5 mM $HAuCl_4$	1 M NaOH or 1 M HCl	50 mM AA; 80 °C; pH 7.1–7.8	Aggregation		The characterization of optical properties, size, and size distributions (DLS, TEM) were done only for His.
		15	10 mL 1 mM $HAuCl_4$	KOH	0.5 mL 100 mM AA + 9.5 mL H_2O; boiling	25.9 ± 0.6 nm (TEM) 46.0 ± 0.2 nm (DLS) spherical		
		16	0.4 mL 20 mg/mL $HAuCl_4$	0.6 mL 0.5 M NaOH	4 mL 0.1 M AA; 37 °C; 6 h	2.5 nm (TEM) spherical		
**9**	**Pro**	1	0.1 mM $HAuCl_4$	No	0.7 mM AA; R.T.; 15-24 h	N.A.	Reduction followed by agglomeration.	The authors stated that a pinkish-red AuNPs solution was formed, but no SEM or UV-vis spectra were provided. Reactions were carried out in µL-scale and, in some cases, with the addition of DMSO.
		2	10 mL 0.01% (w/v) $HAuCl_4$	No	10 mL 1.0 mM AA; boiling	No reduction		The authors used 0.1-5.0 mM concentrations of AA. We used for comparison results of syntheses for AA

								concentration 1.0 mM, for which the largest number of used AA gave stable nanoparticles. There is no complete information on AuNPs characterization results.
		3	0.5 mM $HAuCl_4$	1 M NaOH or 1 M HCl	50 mM AA; 80 °C; pH 7.1–7.8	Aggregation		The characterization of optical properties, size, and size distributions (DLS, TEM) were done only for His.
		17	8.0 mL 3.0 mM $HAuCl_4$	No	2.0 mL 10.0 M AA; boiling for 10 min or 25 °C for 48 h	Particles with ultra-fine dimensions		There are no particles visible on TEM images.
**10**	**Cys**	1	0.1 mM $HAuCl_4$	No	0.7 mM AA; R.T.; >336 h	No reduction	No reduction. White precipitate – Sulfur.	
		2	10 mL 0.01% (w/v) $HAuCl_4$	No	10 mL 1.0 mM AA; boiling	No reduction		The authors used 0.1-5.0 mM concentrations of AA. We used for comparison results of syntheses for AA concentration 1.0 mM, for which the largest number of used AA gave stable nanoparticles. There is no complete information on AuNPs characterization results.
		3	0.5 mM $HAuCl_4$	1 M NaOH or 1 M HCl	50 mM AA; 80 °C; pH 7.1–7.8	No reduction		The characterization of optical properties, size, and size distributions (DLS, TEM) were done only for His.
		18	2 mL 0.5 mM $HAuCl_4$	No	3 mL 0.5mM AA; R.T.; 6 h	78 ± 3 nm at pH 6.0<br>55 ± 3 nm at pH 10.0 (TEM) spherical		
**11**	**Gln**	1	0.1 mM $HAuCl_4$	No	0.7 mM AA; R.T.; 15-24 h	N.A.	Reduction followed by aggregation.	The authors stated that a pinkish-red AuNPs solution was formed, but no SEM or UV-vis spectra were provided. Reactions were carried out in µL-scale

								and, in some cases, with the addition of DMSO.
		2	10 mL 0.01% (w/v) $HAuCl_4$	No	10 mL 1.0 mM AA; boiling	No reduction		The authors used 0.1-5.0 mM concentrations of AA. We used for comparison results of syntheses for AA concentration 1.0 mM, for which the largest number of used AA gave stable nanoparticles. There is no complete information on AuNPs characterization results.
		3	0.5 mM $HAuCl_4$	1 M NaOH or 1 M HCl	50 mM AA; 80 °C; pH 7.1–7.8	Aggregation		The characterization of optical properties, size, and size distributions (DLS, TEM) were done only for His.
**12**	**Asn**	1	0.1 mM $HAuCl_4$	No	0.7 mM AA; R.T.; 15-24 h	N.A.	13.3 nm and 8.5 nm (SEM) spherical	The authors stated that a pinkish-red AuNPs solution was formed, but no SEM or UV-vis spectra were provided. Reactions were carried out in µL-scale and, in some cases, with the addition of DMSO.
		2	10 mL 0.01% (w/v) $HAuCl_4$	No	10 mL 1.0 mM AA; boiling	~50 nm (DLS) ~10 nm (TEM) spherical		The authors used 0.1-5.0 mM concentrations of AA. We used for comparison results of syntheses for AA concentration 1.0 mM, for which the largest number of used AA gave stable nanoparticles. There is no complete information on AuNPs characterization results.
		3	0.5 mM $HAuCl_4$	1 M NaOH or 1 M HCl	25 mM AA; 80 °C; pH 7.1–7.8	N.A. but stable colloids formed		The characterization of optical properties, size, and size distributions (DLS, TEM) were done only for His.
		19	1 mL 100 mM $HAuCl_4$	2.5 mM and 5 mM of NaOH	1-15 mM AA in 99 mL of water; 70 °C; 1-8 h	13.5 ± 3.7 nm (TEM) spherical		Reverse order of reagents addition - $HAuCl_4$ added to AA.

**13**	**Tyr**	1	0.1 mM $HAuCl_4$	No	0.7 mM AA; R.T.; 1-3 h	N.A.	100 nm <<br>(SEM)<br>Irregular	The authors stated that a pinkish-red AuNPs solution was formed, but no SEM or UV-vis spectra were provided. Reactions were carried out in µL-scale and, in some cases, with the addition of DMSO.
		2	10 mL 0.01% (w/v) $HAuCl_4$	No	10 mL 1.0 mM AA; boiling	~200 nm (DLS)<br>~100 nm (TEM)<br>irregular		The authors used 0.1-5.0 mM concentrations of AA. We used for comparison results of syntheses for AA concentration 1.0 mM, for which the largest number of used AA gave stable nanoparticles. There is no complete information on AuNPs characterization results.
		3	0.5 mM $HAuCl_4$	1 M NaOH or 1 M HCl	25 mM AA; 80 °C; pH 7.1–7.8	N.A. but stable colloids formed		The characterization of optical properties, size, and size distributions (DLS, TEM) were done only for His.
		8	0.01% $HAuCl_4$	No	0.5 mg/mL AA; 25 °C; 12 h	~ 100 nm (TEM)<br>spherical and rod-shaped		Short communication with not too many experimental details.
		9	2 mL 5 mM HAuCl4	1 mL KOH	5 mL 10 mM AA; 92 mL water; boiling; reverse order of reagents addition	9.9 nm (TEM)<br>27 nm (DLS)<br>spherical		Solution of $HAuCl_4$ was added to solution of AA and KOH in water.
		20	0.1 mM $HAuCl_4$	1 mM KOH	0.1 mM AA; boiling	30 nm (TEM)<br>spherical		
		21	5 mL 5 mM $HAuCl_4$	HCl<br>2 M NaCl	5 mL 1 mg/L AA; 80 °C; pH=1; 5 days	microwires		
		22	0.034 mM $KAuBr_4$	KOH	0.15 mM AA; R.T.; 20 min	5-40 nm (TEM)<br>spherical		

**14**	**Ser**	1	0.1 mM $HAuCl_4$	No	0.7 mM AA; R.T.; 36-48 h	N.A.	68.6 nm (SEM) spherical	The authors stated that a pinkish-red AuNPs solution was formed, but no SEM or UV-vis spectra were provided. Reactions were carried out in µL-scale and, in some cases, with the addition of DMSO.
		2	10 mL 0.01% (w/v) $HAuCl_4$	No	10 mL 1.0 mM AA; boiling	~120 nm (DLS) ~60 nm (TEM) spherical		The authors used 0.1-5.0 mM concentrations of AA. We used for comparison results of syntheses for AA concentration 1.0 mM, for which the largest number of used AA gave stable nanoparticles. There is no complete information on AuNPs characterization results.
		3	0.5 mM $HAuCl_4$	1 M NaOH or 1 M HCl	50 mM AA; 80 °C; pH 7.1–7.8	N.A. but stable colloids formed		The characterization of optical properties, size, and size distributions (DLS, TEM) were done only for His.
		4	$HAuCl_4$ (Au^{3+} 0.05 mg/mL)	0.5 mol/L NaOH	AA:Au^{3+} 10:1 (w/w); R.T.; 0.5 h in dark then 4 h under UV (365 nm); pH 10	16 ± 2 nm (TEM) 15 ± 1 nm (DLS) spherical		After irradiation $HAuCl_4$/Ser solution changed its color to wine-red.
**15**	**Thr**	1	0.1 mM $HAuCl_4$	No	0.7 mM AA; R.T.; 48-72 h	N.A.	79.2 nm and 63.8 nm (SEM) spherical	The authors stated that a pinkish-red AuNPs solution was formed, but no SEM or UV-vis spectra were provided. Reactions were carried out in µL-scale and, in some cases, with the addition of DMSO.
		2	10 mL 0.01% (w/v) $HAuCl_4$	No	10 mL 1.0 mM AA; boiling	~110 nm (DLS) ~60 nm (TEM) spherical		The authors used 0.1-5.0 mM concentrations of AA. We used for comparison results of syntheses for AA concentration 1.0 mM, for which the largest number of used AA gave stable nanoparticles. There is no complete

								information on AuNPs characterization results.
		3	0.5 mM $HAuCl_4$	1 M NaOH or 1 M HCl	50 mM AA; 80 °C; pH 7.1–7.8	No reduction		The characterization of optical properties, size, and size distributions (DLS, TEM) were done only for His.
**16**	**Hyp**	Our studies					63.5 nm and 52.0 nm (SEM) nanoclusters	There were no other studies than ours on using Hyp to reduce gold salts.
**17**	**Glu**	1	0.1 mM $HAuCl_4$	No	0.7 mM AA; R.T.; 15-24 h	N.A.	27.0 nm and 11.2 nm (SEM) spherical	The authors stated that a pinkish-red AuNPs solution was formed, but no SEM or UV-vis spectra were provided. Reactions were carried out in µL-scale and, in some cases, with the addition of DMSO.
		2	10 mL 0.01% (w/v) $HAuCl_4$	No	10 mL 1.0 mM AA; boiling	~50 nm (DLS) ~20 nm (TEM) spherical		The authors used 0.1-5.0 mM concentrations of AA. We used for comparison results of syntheses for AA concentration 1.0 mM, for which the largest number of used AA gave stable nanoparticles. There is no complete information on AuNPs characterization results.
		3	0.5 mM $HAuCl_4$	1 M NaOH or 1 M HCl	25 mM AA; 80 °C; pH 7.1–7.8	Aggregation		The characterization of optical properties, size, and size distributions (DLS, TEM) were done only for His.
		6	5 mM $HAuCl_4$	No	25 mM AA; boiling	5-20 nm (TEM) 26 nm (DLS) spherical		Studies were done for different concentrations of $HAuCl_4$ and AA.
		23	0.01% $HAuCl_4$	No	1-20 mM AA; boiling	40 ± 5 nm for 10 mM AA 40 ± 2 nm for 15 mM AA		

						40 ± 15 nm for 20 mM AA (TEM) spherical		
**18**	**Asp**	1	0.1 mM $HAuCl_4$	No	0.7 mM AA; R.T.; 3-15 h	N.A.	18.8 nm and 9.8 nm (SEM) spherical	The authors stated that a pinkish-red AuNPs solution was formed, but no SEM or UV-vis spectra were provided. Reactions were carried out in µL-scale and, in some cases, with the addition of DMSO.
		2	10 mL 0.01% (w/v) $HAuCl_4$	No	10 mL 1.0 mM AA; boiling	~50 nm (DLS) ~20-30 nm (TEM) spherical		The authors used 0.1-5.0 mM concentrations of AA. We used for comparison results of syntheses for AA concentration 1.0 mM, for which the largest number of used AA gave stable nanoparticles. There is no complete information on AuNPs characterization results.
		3	0.5 mM $HAuCl_4$	1 M NaOH or 1 M HCl	25 mM AA; 80 °C; pH 7.1–7.8	N.A. but stable colloids formed		The characterization of optical properties, size, and size distributions (DLS, TEM) were done only for His.
		4	$HAuCl_4$ (Au^{3+} 0.05 mg/mL)	0.5 mol/L NaOH	AA:Au^{3+} 10:1 (w/w); R.T.; 0.5 h in dark then 4 h under UV (365 nm); pH 10	16 ± 4 nm (TEM) 21 ± 2 nm (DLS) spherical		After irradiation, $HAuCl_4$/Asp solution changed its color to wine-red.
		8	0.01% $HAuCl_4$	No	0.5 mg/mL AA; 25 °C; 12 h	> 100 nm (TEM of spherical) spherical and plate-shape		Short communication with limited description of experimental conditions.
		24	90 mL 0.1 mM $HAuCl_4$	No	10 mL 10.0 mM, 1.0 mM, and 0.5 mM AA; boiling	monodispersed spherical 1.0 mM AA 24 ± 3 nm		Control experiments were performed as using the same conditions for the amino acids, valine and lysine, however no reduction of $HAuCl_4$ was observed.

						variations of size and shape<br>10.0 mM AA<br>29 ± 3 nm<br>0.5 mM AA<br>42 nm<br>(TEM)		
		25	0.05-1.0<br>mM $HAuCl_4$	No	2.50, 0.50, and 0.25 mM AA; R.T.;<br>> 4 h	Nanospheres,<br>Nanoplates,<br>Nanoribbons,<br>and Nanowires		Authors studied effect of reagents concentrations on reactions outcome.
**19**	**His**	1	0.1 mM $HAuCl_4$	No	0.7 mM AA; R.T.;<br>72-336 h	N.A.	50 nm <<br>(SEM)<br>Irregular	The authors stated that a pinkish-red AuNPs solution was formed, but no SEM or UV-vis spectra were provided. Reactions were carried out in µL-scale and, in some cases, with the addition of DMSO.
		2	10 mL 0.01%<br>(w/v) $HAuCl_4$	No	10 mL 1.0 mM AA;<br>boiling	No reduction		The authors used 0.1-5.0 mM concentrations of AA. We used for comparison results of syntheses for AA concentration 1.0 mM, for which the largest number of used AA gave stable nanoparticles. There is no complete information on AuNPs characterization results.
		3	0.5 mM $HAuCl_4$	1 M NaOH<br>or 1 M HCl	50 mM AA; 80 °C;<br>pH 7.1–7.8	4-7 nm (TEM)<br>spherical		The characterization of optical properties, size, and size distributions (DLS, TEM) were done only for His.
		14	5.078 mM<br>$HAuCl_4$	No	0.05 M AA; 37 °C<br>then 25 °C; 72 h	No reduction		Formation of self-assembled coordination polymeric structures.
		26	1.0 mL 10 mM<br>$HAuCl_4$		3.0 mL 100 mM<br>AA; 30 °C; 3 h	2.2 ± 0.6 nm<br>(TEM)<br>spherical		Glutathione was added to product of $HAuCl_4$ reduction by AA.

**20**	**Lys**	1	0.1 mM $HAuCl_4$	No	0.7 mM AA; R.T.; 24-30 h	N.A.	50 nm < (SEM) Irregular	The authors stated that a pinkish-red AuNPs solution was formed, but no SEM or UV-vis spectra were provided. Reactions were carried out in µL-scale and, in some cases, with the addition of DMSO.
		2	10 mL 0.01% (w/v) $HAuCl_4$	No	10 mL 1.0 mM AA; boiling	No reduction		The authors used 0.1-5.0 mM concentrations of AA. We used for comparison results of syntheses for AA concentration 1.0 mM, for which the largest number of used AA gave stable nanoparticles. There is no complete information on AuNPs characterization results.
		3	0.5 mM $HAuCl_4$	1 M NaOH or 1 M HCl	50 mM AA; 80 °C; pH 7.1–7.8	Aggregation		The characterization of optical properties, size, and size distributions (DLS, TEM) were done only for His.
		4	$HAuCl_4$ (Au^{3+} 0.05 mg/mL)	0.5 mol/L NaOH	AA:Au^{3+} 10:1 (w/w); R.T.; 0.5 h in dark then 4 h under UV (365 nm); pH 10	11 ± 4 nm (TEM) 15 ± 3 nm (DLS) spherical		After irradiation $HAuCl_4$/Lys solution changed its color to violet.
		8	0.01% $HAuCl_4$	No	0.5 mg/mL AA; 25 °C; 12 h	6 ± 2 nm (TEM) spherical		Short communication with not too many experimental details.
**21**	**Arg**	1	0.1 mM $HAuCl_4$	No	0.7 mM AA; R.T.; 24-30 h	N.A.	Reduction followed by agglomeration. Formation of large irregular structures.	The authors stated that a pinkish-red AuNPs solution was formed, but no SEM or UV-vis spectra were provided. Reactions were carried out in µL-scale and, in some cases, with the addition of DMSO.
		2	10 mL 0.01% (w/v) $HAuCl_4$	No	10 mL 1.0 mM AA; boiling	Incomplete reduction		The authors used 0.1-5.0 mM concentrations of AA. We used for comparison results of syntheses for AA concentration 1.0 mM, for which the

								largest number of used AA gave stable nanoparticles. There is no complete information on AuNPs characterization results.
		3	0.5 mM $HAuCl_4$	1 M NaOH or 1 M HCl	50 mM AA; 80 °C; pH 7.1–7.8	No reduction		The characterization of optical properties, size, and size distributions (DLS, TEM) were done only for His.
		8	0.01% $HAuCl_4$	No	0.5 mg/mL AA; 25 °C; 12 h	10 ± 5 nm (TEM) Spherical with wide distribution		Short communication with not too many experimental details.
		22	0.034 mM $KAuBr_4$	KOH	0.15 mM AA; R.T.; > 40 h	15-50 nm (TEM) irregular		

Abbreviations:

AA – Amino Acid

N.A. – not available

R.T. – room temperature

w/w – weight per unit weight (weight-to-weight ratio)

Tables S2. The pKa, pKb, pKx, and pI values and solubility s [g/L H_2O] of the amino acids used in the studies as reducing and capping agents.[27-31]

#	Amino acid	pK_a (-COOH)	pK_b ($-NH_3^+$)	pK_x (side chain)	pI	s [g/L H_2O]
1.	L-glycine (Gly)	2.34	9.60	-	5.97	251
2.	L-alanine (Ala)	2.34	9.69	-	6.02	167
3.	L-valine (Val)	2.32	9.62	-	5.97	58
4.	L-leucine (Leu)	2.36	9.60	-	5.98	23
5.	L-isoleucine (Ile)	2.36	9.60	-	6.02	34
6.	L-phenylalanine (Phe)	1.83	9.13	-	5.48	29
7.	L-tryptophan (Trp)	2.83	9.39	-	5.88	12
8.	L-methionine (Met)	2.28	9.21	-	5.75	56
9.	L-proline (Pro)	1.99	10.60	-	6.30	1622
10.	L-cysteine (Cys)	1.96	10.28	8.18 (-SH)	5.02	277
11.	L-glutamine (Gln)	2.17	9.13	-	5.65	42
12.	L-asparagine (Asn)	2.02	8.80	-	5.41	25
13.	L-tyrosine (Tyr)	2.20	9.11	10.07 (-OH)	5.65	0.5
14.	L-serine (Ser)	2.21	9.15	-	5.68	422
15.	L-threonine (Thr)	2.09	9.10	-	6.53	97
16.	L-hydroxyproline (Hyp)	1.82	9.65	N.A.	N.A.	361
17.	L-glutamic acid (Glu)	2.19	9.67	4.25 (-COOH)	3.22	8.57
18.	L-aspartic acid (Asp)	1.88	9.60	3.65 (-COOH)	2.87	5
19.	L-histidine (His)	1.82	9.17	6.00 (imidazolium)	7.58	43
20.	L-lysine (Lys)	2.18	8.95	10.53 ($-NH_3^+$)	9.74	6
21.	L-arginine (Arg)	2.17	9.04	12.48 ($-NH_3^+$)	10.76	181

N.A. – not available

Tables S3. Comparison of obtained results with other relevant studies.[1-3]

#	Amino acid	Our studies	Ref [3][a]	Ref [2][b]	Ref [1][c]
1.	L-glycine (Gly)	Purple solution	Purple solution	No reduction	Red solution
2.	L-alanine (Ala)	Red solution	Purple solution	No reduction	Red solution
3.	L-valine (Val)	Red solution	Red solution	Red solution	Red solution
4.	L-leucine (Leu)	Red solution	Aggregation/Precipitation	Red solution	Red solution
5.	L-isoleucine (Ile)	Red solution	Aggregation/Precipitation	No reduction	Red solution
6.	L-phenylalanine (Phe)	Red solution	Purple solution	Red solution	Red solution
7.	L-tryptophan (Trp)	Purple solution	Purple solution	Aggregation/Precipitation	Red solution
8.	L-methionine (Met)	Purple solution	Aggregation/Precipitation	No reduction	Red solution
9.	L-proline (Pro)	Aggregation/Precipitation	Aggregation/Precipitation	No reduction	Red solution
10.	L-cysteine (Cys)	No reduction	No reduction	No reduction	No reduction
11.	L-glutamine (Gln)	Aggregation/Precipitation	Aggregation/Precipitation	No reduction	Red solution
12.	L-asparagine (Asn)	Red solution	Red solution	Red solution	Red solution
13.	L-tyrosine (Tyr)	Purple solution	Purple solution	Red solution	Red solution
14.	L-serine (Ser)	Purple solution	Purple solution	Red solution	Red solution
15.	L-threonine (Thr)	Purple solution	No reduction	Red solution	Red solution
16.	L-hydroxyproline (Hyp)	Blue solution	Not investigated	Not investigated	Not investigated
17.	L-glutamic acid (Glu)	Red solution	Aggregation/Precipitation	Red solution	Red solution
18.	L-aspartic acid (Asp)	Red solution	Purple solution	Red solution	Red solution
19.	L-histidine (His)	Purple solution	Purple solution	No reduction	Red solution
20.	L-lysine (Lys)	Purple solution	Aggregation/Precipitation	No reduction	Red solution
21.	L-arginine (Arg)	Aggregation/Precipitation	No reduction	No reduction	Red solution

[a] Based on AuNPs solutions images in Figure 1; [b] Based on results for c=1.0 mM shown in Figure 1; [c] Based on Table S1 and description in article. No TEM/SEM data, images of AuNPs solutions or UV-vis spectra were provided for AuNPs made with amino acids.

	Red solution
	Purple solution
	Blue solution
	Aggregation/Precipitation
	No reduction
	Not investigated

Figure S1. SEM images of AuNPs synthesized using amino acids **1-8** and **12-20**. Images are enlarged compared to Figure 3 to better visualize morphology of obtained AuNPs.

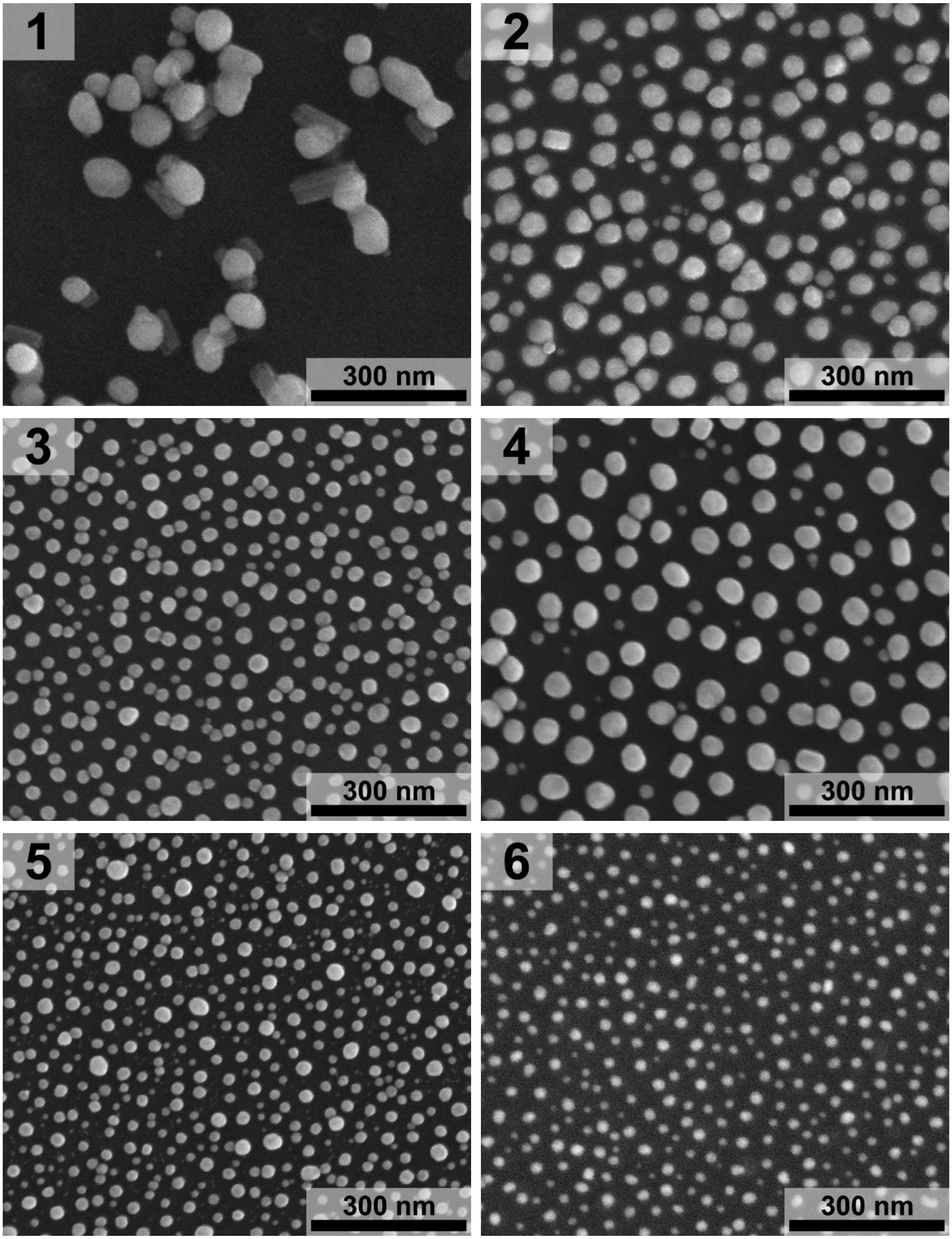

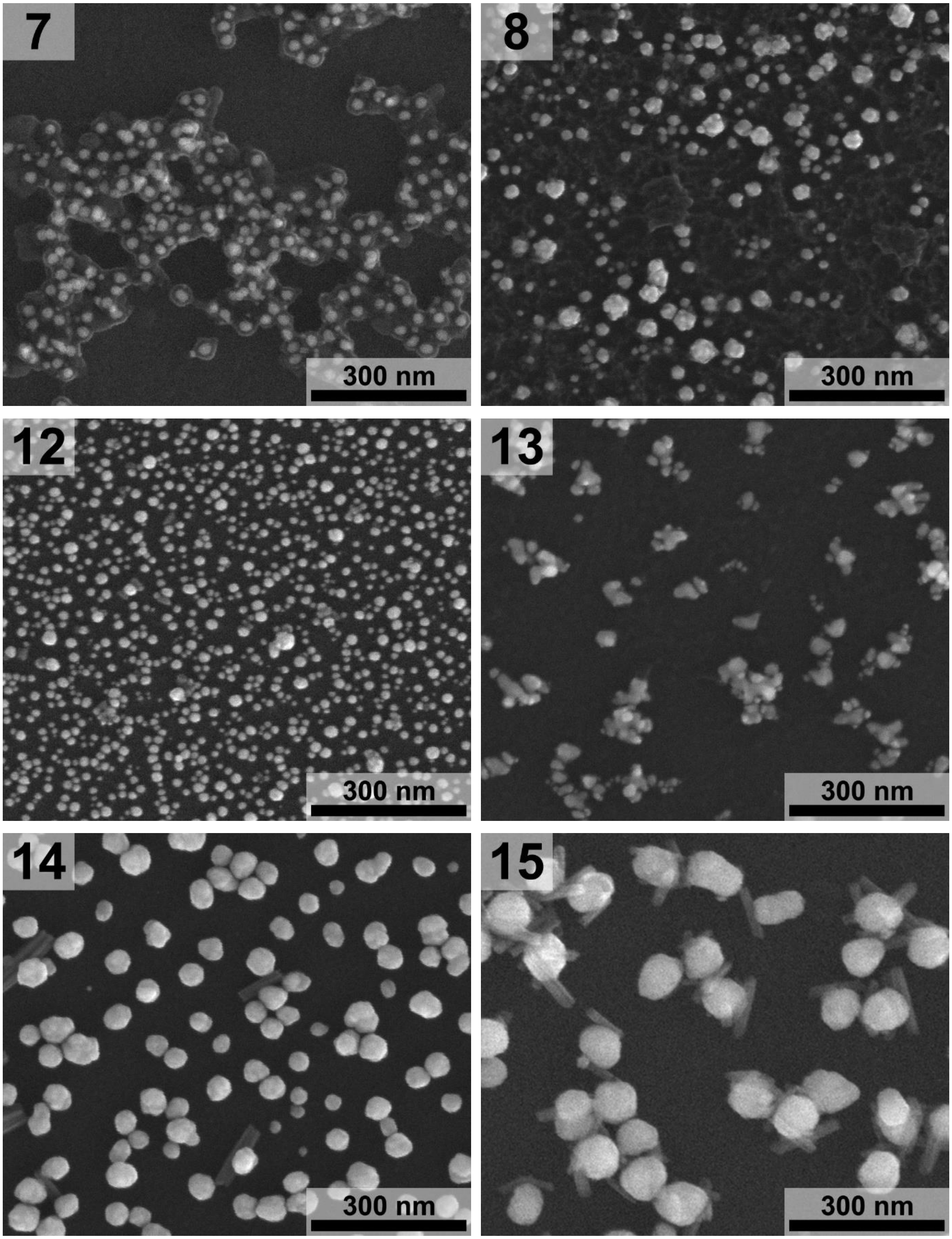
7
300 nm
8
300 nm
12
300 nm
13
300 nm
14
300 nm
15
300 nm

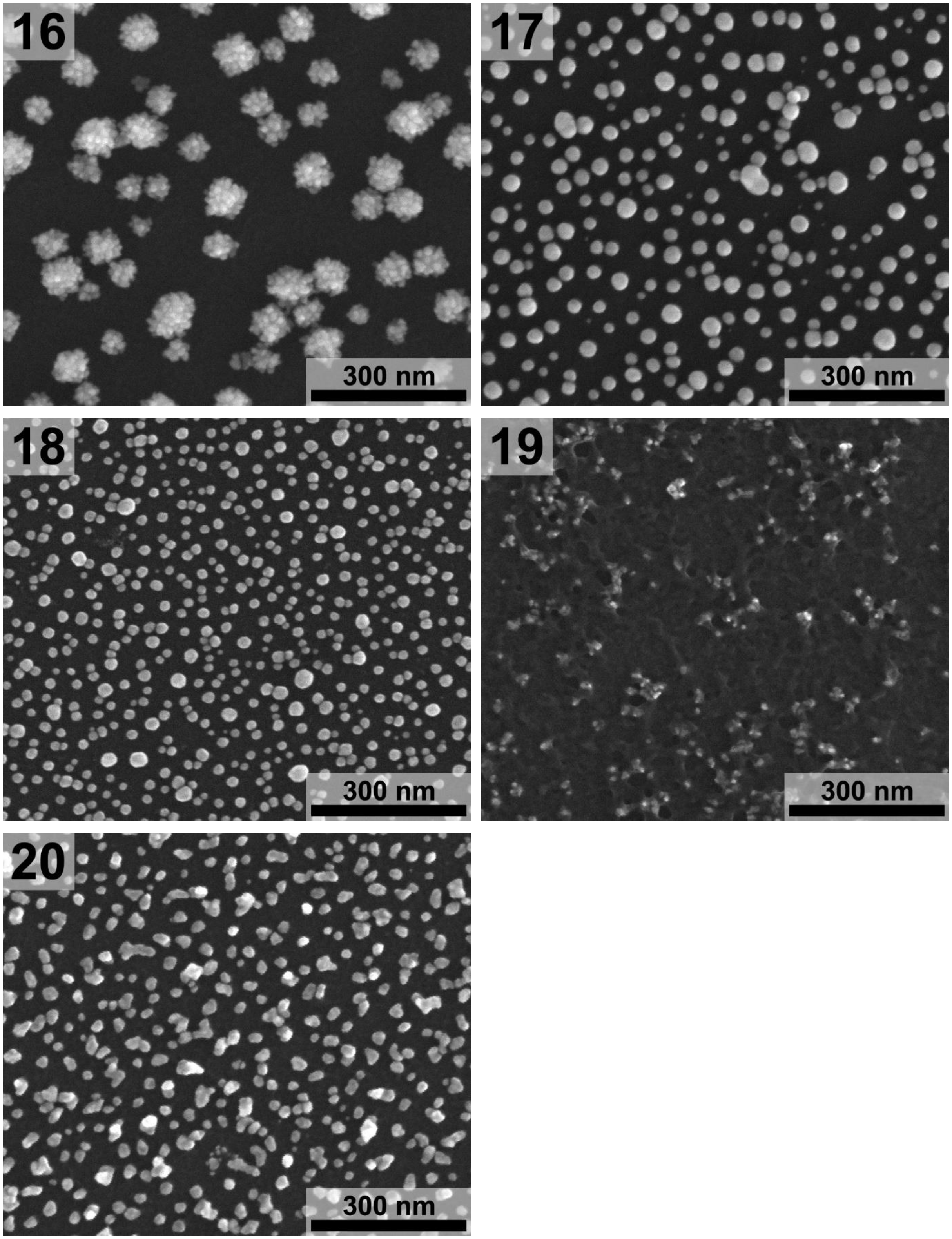
16
300 nm
17
300 nm
18
300 nm
19
300 nm
20
300 nm

Figure S2. Size distributions of AuNPs synthesized using amino acids **1-8**, **12**, and **14-18** determined by SEM images analysis and number-weighted size distribution obtained by DCS. In comparison to Figure 2, charts were rescaled and multimodal distributions were deconvolved.

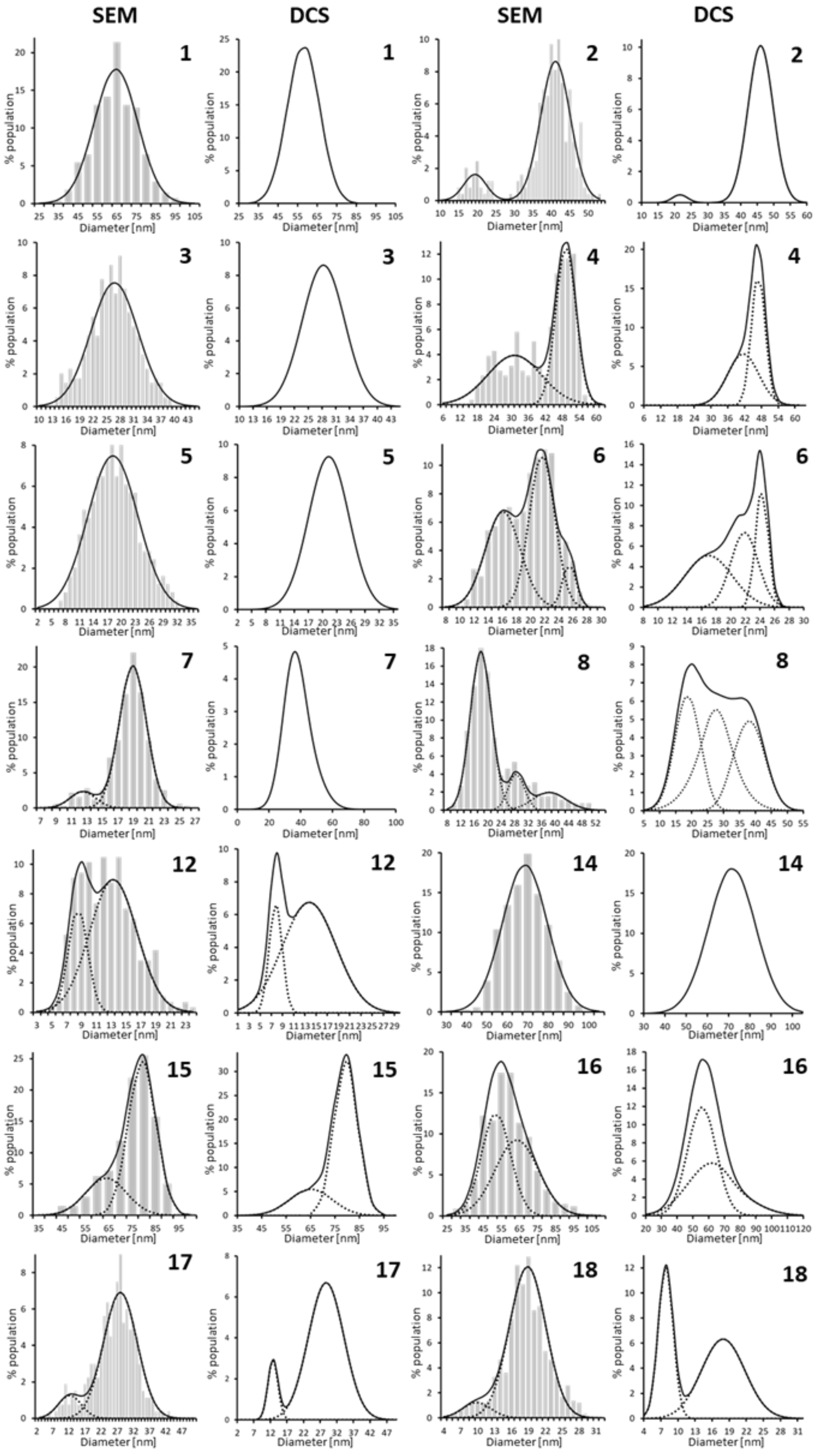

Figure S3. Stability of AuNPs synthesized using reducing agents **2-8** and **12-20** indicated by absorption spectra measured one day after synthesis (green), after 4 weeks' storage (blue) and after 8 weeks' storage (red). AuNPs synthesized with L-glycine **1** were unstable over time.

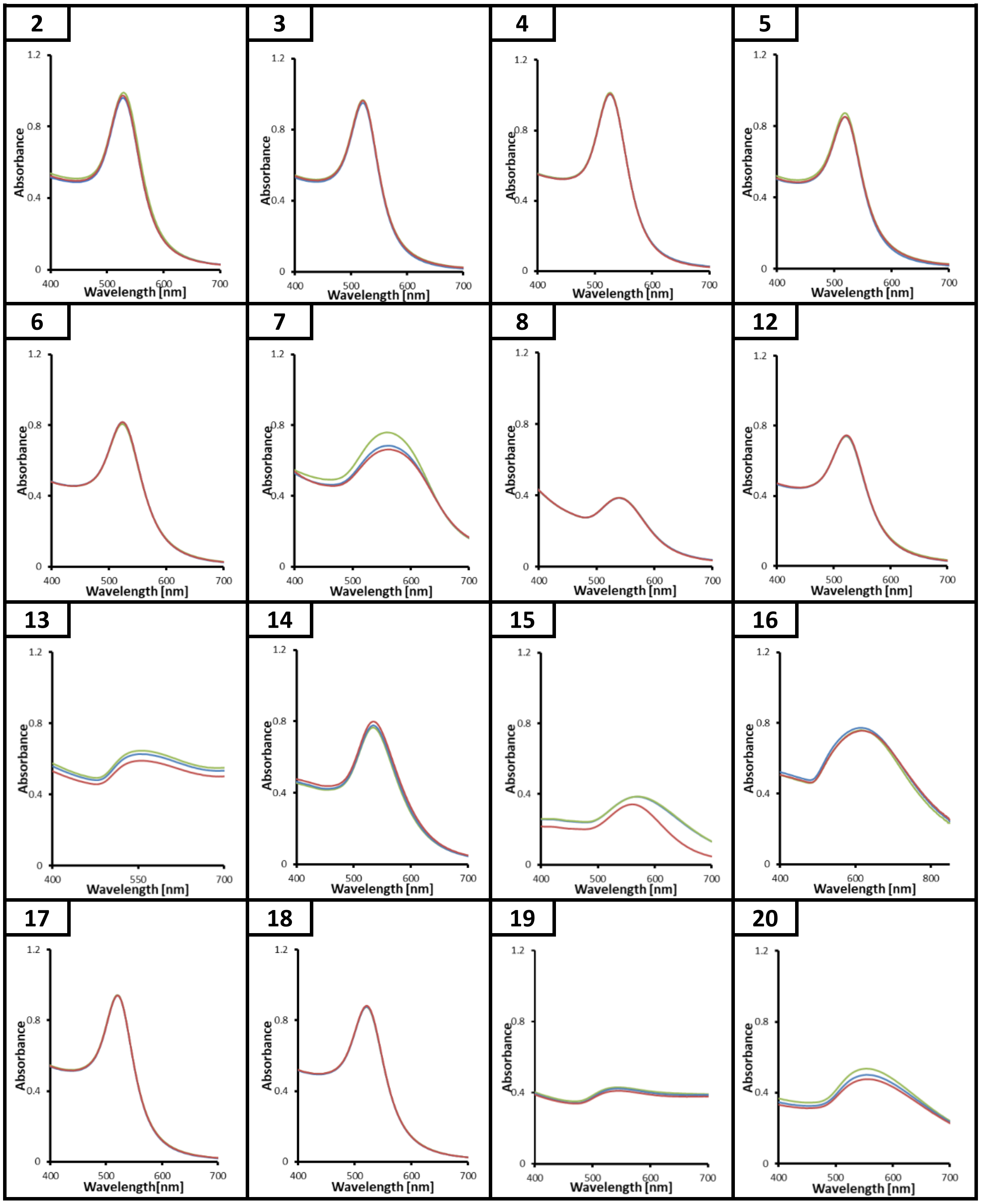

References

1. Tan, Y.N.; Lee, J.Y.; Wang, D.I.C. Uncovering the Design Rules for Peptide Synthesis of Metal Nanoparticles. *J. Am. Chem. Soc.* **2010**, *132*, 5677-5686. doi: 10.1021/ja907454f.

2. Wangoo, N.; Kaur, S.; Bajaj, M.; Jain, D.V.S.; Sharma, R.K. One Pot, Rapid and Efficient Synthesis of Water Dispersible Gold Nanoparticles Using Alpha-Amino Acids. *Nanotech.* **2014**, *25*, 435608. doi:10.1088/0957-4484/25/43/435608.

3. Maruyama, T.; Fujimoto, Y.; Maekawa, T. Synthesis of Gold Nanoparticles Using Various Amino Acids. *J. Colloid Interface Sci.* **2015**, *447*, 254-257. doi:10.1016/j.jcis.2014.12.046.

4. Cai, H.; Yao, P. Gold Nanoparticles With Different Amino Acid Surfaces: Serum Albumin Adsorption, Intracellular Uptake and Cytotoxicity. *Colloids Surf. B: Biointerfaces.* **2014**, *123*, 900-906. doi:10.1016/j.colsurfb.2014.10.042.

5. Berghian-Grosan, C.; Olenic, L.; Katona, G.; Perde-Schrepler, M.; Vulcu, A. L-Leucine for Gold Nanoparticles Synthesis and Their Cytotoxic Effects Evaluation. *Amino acids* **2014**, *46*, 2545-2552. doi:10.1007/s00726-014-1814-z.

6. Zare, D.; Khoshnevisan, K.; Barkhi, M.; Tahami, H.V. Fabrication of Capped Gold Nanoparticles by Using Various Amino Acids. *J. Exp. Nanosci.* **2014**, *9*, 957-965. doi:10.1080/17458080.2012.752582.

7. Nayak, N.C.; Shin, K. Synthesis of L-Phenylalanine Stabilized Gold Nanoparticles and Their Thermal Stability. *J. Nanosci. Nanotechnol.* **2006**, *6*, 3512-3516. doi:10.1166/jnn.2006.17972.

8. Shao, Y.; Jin, Y.; Dong, S. Synthesis of Gold Nanoplates by Aspartate Reduction of Gold Chloride. *Chem. Commun.* **2004**, *9*, 1104-1105. doi:10.1039/B315732F.

9. Carnovale, C.; Bryant, G.; Shukla, R.; Bansal, V. Identifying Trends in Gold Nanoparticle Toxicity and Uptake: Size, Shape, Capping Ligand, and Biological Corona. *ACS Omega* **2019**, *4*, 242-256. doi:10.1021/acsomega.8b03227.

10. Selvakannan, P.R.; Mandal, S.; Phadtare, S.; Gole, A.; Pasricha, R.; Adyanthaya, S.D.; Sastry, M. Water-Dispersible Tryptophan-Protected Gold Nanoparticles Prepared by the Spontaneous Reduction of Aqueous Chloroaurate Ions by the Amino Acid. *J. Colloid Interface Sci.* **2004**, *269*, 97-102. doi:10.1016/S0021-9797(03)00616-7.

11. Akbarzadeh, A.; Zare, D.; Farhangi, A.; Mehrabi, M.R.; Norouzian, D.; Tangestaninejad, S.; Moghadam, M.; Bararpour, N. Synthesis and Characterization of Gold Nanoparticles by Tryptophane. *Am. J. Appl. Sci.* **2009**, *6*, 691-695. doi:10.3844/ajassp.2009.691.695.

12. Iosin, M.; Baldeck, P.; Astilean. S. Study of Tryptophan Assisted Synthesis of Gold Nanoparticles by Combining UV–Vis, Fluorescence, and SERS Spectroscopy. *J. Nanopart. Res.* **2010**, *12*, 2843-2849. doi:10.1007/s11051-010-9869-6.

13. Sun, J.; Liu, G.; Fu, S.; Cai, F.; Yin, H.; Lv, H.; He, J. Gold Nanoparticles of Multiple Shapes Synthesized in L-Tryptophan Aqueous Solution. *Trans. Tianjin Univ.* **2018**, *24*, 401-414. doi:10.1007/s12209-018-0141-y.

14. Csapó, E.; Ungor, D.; Kele, Z.; Baranyai, P.; Deák, A.; Juhász, Á.; Janovák, L.; Dékány, I. Influence of pH and Aurate/Amino Acid Ratios on the Tunable Optical Features of Gold Nanoparticles and Nanoclusters. *Colloids Surf. A Physicochem. Eng. Asp.* **2017**, *532*, 601-608. doi:10.1016/j.colsurfa.2017.02.047.

15. Laban, B.; Ralević, U.; Petrović, S.; Leskovac, A.; Vasić-Anićijević, D.; Marković, M.; Vasić, V. Green Synthesis and Characterization of Nontoxic L-Methionine Capped Silver and Gold Nanoparticles. *J. Inorg. Biochem.* **2020**, *204*, 110958. doi:10.1016/j.jinorgbio.2019.110958.

16. Deng, H.H.; Zhang, L.N.; He, S.B.; Liu, A.L.; Li, G.W.; Lin, X.H.; Xia, X.H.; Chen. W. Methionine-Directed Fabrication of Gold Nanoclusters with Yellow Fluorescent Emission for Cu^{2+} Sensing. *Biosens. Bioelectron.* **2015**, *65*, 397-403. doi:10.1016/j.bios.2014.10.071.

17. Mu, X.; Qi, L.; Dong, P.; Qiao, J.; Hou, J.; Nie, Z.; Ma, H. Facile One-Pot Synthesis of L-Proline-Stabilized Fluorescent Gold Nanoclusters and its Application as Sensing Probes for Serum Iron. *Biosens. Bioelectron.* **2013**, *49*, 249-255. doi:10.1016/j.bios.2013.05.019.

18. Ma, Z.; Han, H. One-Step Synthesis of Cystine-Coated Gold Nanoparticles in Aqueous Solution. *Colloids Surf. Physicochem. Eng. Aspects* **2008**, *317*, 229-233. doi:10.1016/j.colsurfa.2007.10.018.

19. Garg, N.; Bera, S.; Rastogi, L.; Ballal, A.; Balaramakrishna, M.V. Synthesis and Characterization of L-Asparagine Stabilised Gold Nanoparticles: Catalyst for

Degradation of Organic Dyes. *Spectrochim. Acta A Mol. Biomol. Spectrosc.* **2020**, *232*, 118126. doi:10.1016/j.saa.2020.118126.

20. Selvakannan, P.R.; Ramanathan, R.; Plowman, B.J.; Sabri, Y.M.; Daima, H.K.; O'Mullane, A.P.; Bansal, V.; Bhargava, S.K. Probing the Effect of Charge Transfer Enhancement in Off Resonance Mode SERS via Conjugation of the Probe Dye Between Silver Nanoparticles and Metal Substrates. *Phys. Chem. Chem. Phys.* **2013**, *15*, 12920-12929. doi: 10.1039/C3CP51646F.

21. Lv, L.; Han, X.; Wu, X.; Li, C. The Synthesis of High-Aspect-Ratio Au Microwires with a Biomolecule for Electrochemical Sensing. *Chem. Commun.* **2020**, *56*, 743-746. doi: 10.1039/C9CC06523G.

22. Bhargava, S.K.; Booth, J.M.; Agrawal, S.; Coloe, P.; Kar, G. Gold Nanoparticle Formation During Bromoaurate Reduction by Amino Acids. *Langmuir* **2005**, *21*, 5949-5956. doi:10.1021/la050283e.

23. Wangoo, N.; Bhasin, K.K.; Mehta, S.K.; Suri, C.R. Synthesis and Capping of Water-Dispersed Gold Nanoparticles by an Amino Acid: Bioconjugation and Binding Studies. *J. Colloid Interface Sci.* **2008**, *323*, 247-254. doi:10.1016/j.jcis.2008.04.043.

24. Mandal, S.; Selvakannan, P.R.; Phadtare, S.; Pasricha, R.; Sastry, M. Synthesis of a Stable Gold Hydrosol by the Reduction of Chloroaurate Ions by the Amino Acid, Aspartic Acid. *J. Chem. Sci.* **2002**, *114*, 513-520. doi:10.1007/BF02704195.

25. Tan, Y.N.; Lee, J.Y.; Wang, D.I.C. Aspartic Acid Synthesis of Crystalline Gold Nanoplates, Nanoribbons, and Nanowires in Aqueous Solutions. *J. Phys. Chem. C* **2008**, *112*, 5463-5470. doi:10.1021/jp800501k.

26. Bhamore, J.R.; Gul, A.R.; Chae, W.S.; Kim, K.W.; Lee, J.S.; Park, H.; Kailasa, S.K.; Park, T.J. One-Pot Fabrication of Amino Acid and Peptide Stabilized Gold Nanoclusters for the Measurement of the Lead in Plasma Samples Using Chemically Modified Cellulose Paper. *Sens. Actuators B Chem.* **2020**, *322*, 128603. doi:10.1016/j.snb.2020.128603.

27. Lide, D.R. CRC Handbook of Chemistry and Physics, CRC Press Boca Raton, 1997-1998, 7-1.

28. https://pubchem.ncbi.nlm.nih.gov/compound/Cysteine#section=Solubility (accessed on December 11, 2022).

29. https://pubchem.ncbi.nlm.nih.gov/compound/Hydroxyproline#section=Solubility (accessed on December 11, 2022).

30. https://pubchem.ncbi.nlm.nih.gov/compound/Glutamic-acid#section=Solubility (accessed on December 11, 2022).

31. Fleck, M.; Petrosyan. A.M. *Salts of amino acids. Crystallization, Structure and Properties.* Springer, Dordrecht, Netherlands, 2014.
